# The temporal balance between self-renewal and differentiation of human neural stem cells requires the amyloid precursor protein

**DOI:** 10.1126/sciadv.add5002

**Published:** 2023-06-16

**Authors:** Khadijeh Shabani, Julien Pigeon, Marwan Benaissa Touil Zariouh, Tengyuan Liu, Azadeh Saffarian, Jun Komatsu, Elise Liu, Natasha Danda, Mathilde Becmeur-Lefebvre, Ridha Limame, Delphine Bohl, Carlos Parras, Bassem A. Hassan

**Affiliations:** ^1^ Institut du Cerveau–Paris Brain Institute–ICM, Sorbonne Université, INSERM, CNRS, Hôpital Pitié-Salpêtrière, Paris, France.; ^2^ Scipio bioscience, iPEPS-ICM, Hôpital Pitié-Salpêtrière, Paris, France.; ^3^ Genetics and Foetopathology, Centre Hospitalier Regional d’Orleans–Hôpital de la Source, Orleans, France.

## Abstract

Neurogenesis in the developing human cerebral cortex occurs at a particularly slow rate owing in part to cortical neural progenitors preserving their progenitor state for a relatively long time, while generating neurons. How this balance between the progenitor and neurogenic state is regulated, and whether it contributes to species-specific brain temporal patterning, is poorly understood. Here, we show that the characteristic potential of human neural progenitor cells (NPCs) to remain in a progenitor state as they generate neurons for a prolonged amount of time requires the amyloid precursor protein (APP). In contrast, APP is dispensable in mouse NPCs, which undergo neurogenesis at a much faster rate. Mechanistically, APP cell-autonomously contributes to protracted neurogenesis through suppression of the proneurogenic activator protein–1 transcription factor and facilitation of canonical WNT signaling. We propose that the fine balance between self-renewal and differentiation is homeostatically regulated by APP, which may contribute to human-specific temporal patterns of neurogenesis.

## Teaser

The slow tempo of human cortical neurogenesis requires the Alzheimer's disease Amyloid Precursor Protein.

## INTRODUCTION

The human brain has expanded over 15-fold since our divergence from old world monkeys and 3-fold since our divergence from a common ancestor with chimpanzees (*1*). The cerebral cortex (neocortex) accounts for two-thirds of brain size with enormous diversity in cell type, morphology, connectivity, and function. It is typically structured into six radial layers of cells with highly elaborate neuronal circuits and is responsible for complex cognitive behaviors including sensory perception and skilled motor planning, attention, language, emotion, and consciousness (*2*, *3*). Human cortical neurogenesis begins at around 5 gestational weeks (GW) and is mostly completed by 28 GW (*4*, *5*). An important factor underlying human cortical expansion is an intrinsic capacity of human cortical progenitors to generate very large numbers of neurons over extended periods of time relative to other mammals, while retaining a self-renewing progenitor state for a particularly long time, presumably through species-specific mechanisms that remain poorly understood (*6*, *7*).

The highly conserved class I transmembrane amyloid precursor protein (APP) is broadly expressed throughout nervous system development and has been extensively studied because its proteolytic processing is linked to Alzheimer’s disease (AD) (*8*–*10*), yet its physiological function, especially in humans, is unclear. APP and its homologs in various species are involved in many biological processes such as axonal outgrowth after injury (*11*), endolysosomal pathway (*12*–*14*), stress response after hypoxia/ischemia (*15*, *16*), cell signaling processes, and brain development and plasticity (*17*, *18*). In the worm *Caenorhabditis elegans*, loss of the APP homolog *Apl* is lethal because of molting deficits, while causing mild and low penetrance defects in various aspects of neuronal differentiation, function, and survival in mouse and *Drosophila* (*19*). In humans, point mutations of even a single copy of *APP* cause familial AD (*20*). APP is highly expressed in human telencephalic neurospheres and during the differentiation and migration of cortical neurons (*21*–*23*), suggesting that it may be involved in neural stem cells proliferation, differentiation, and/or maturation (*24*, *25*). In the adult rodent brain, APP is also abundantly expressed in progenitor cells found in the ventricular zone (VZ)–subventricular zone (SVZ) (*26*, *27*), but its loss does not cause obvious deficits in neurogenesis. We generated *APP*-knockout human induced pluripotent stem cells (iPSCs) with two different genetic backgrounds and queried the potential effect on human cortical neurogenesis.

## RESULTS

### Increased generation of human cortical neurons in the absence of APP

**Fig. 1.**
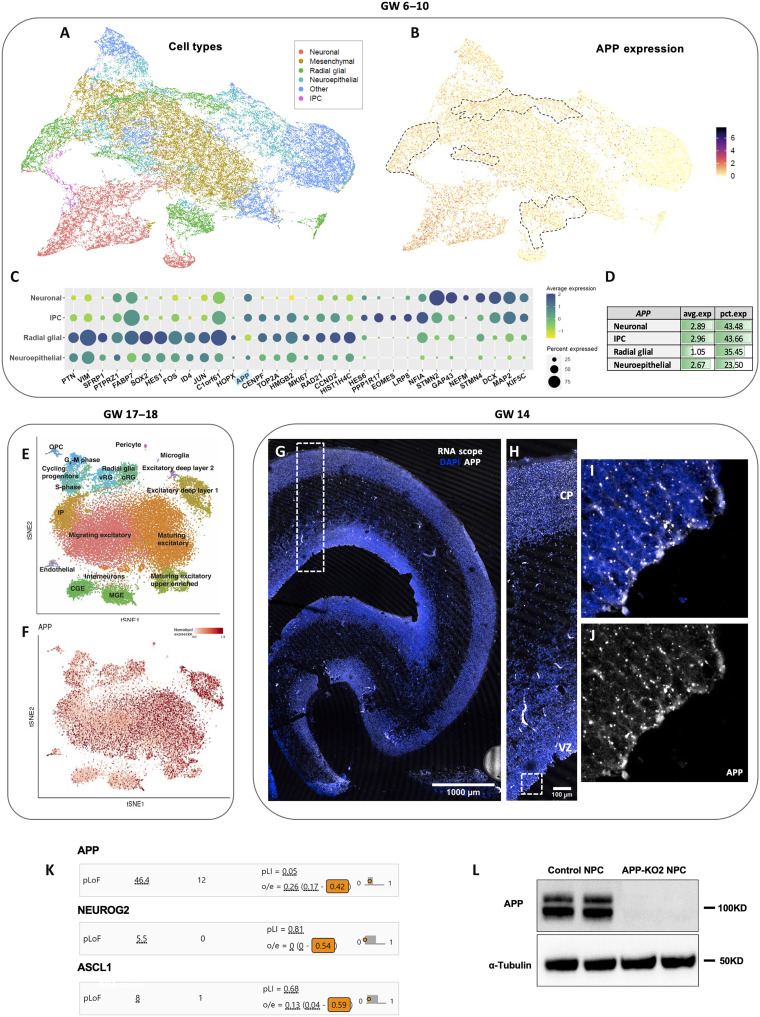
APP expression in the developing human cortex. (**A** to **D**) APP is expressed in all cell types detected by scRNAseq of the human fetal cortex during GW6 to GW10 (*28*) and (**E** and **F**) GW17 to GW18 of development (*22*). (**G** and **H**) APP is expressed in different regions of human developing cortex at GW14 from VZ to CP (scale bars, 1000 and 100 μm). (**I** and **J**) Enrichment of *APP* on apical pole of aRGCs. (**K**) *APP* loss of function results in significant developmental lethality in the human population with a lower observed/expected (LOEUF) score (0.42) compared to two key neurodevelopmental genes such as NEUROG2 (LOEUF = 0.54) and ASCL1 (LOEUF = 0.59). (**L**) APP presence in control iPSC-derived human NPCs was confirmed by Western blot.

To examine *APP* expression during human fetal cortical development, we queried single-cell RNA sequencing (scRNA-seq) data from the developing human cortex at GW6 to GW10 (*28*) (https://cells-test.gi.ucsc.edu/?ds=early-brain) and found that *APP* is present in all six types of cells, with expression detected in 23.50% of neuroepithelial cell (NEC), 35.45% of radial glial cell (RGC), 43.66% of intermediate progenitor cell (IPC) and 43.48% of neurons (Fig. 1, A to D) present in that dataset. Furthermore, this expression appears particularly dynamic as cells transition from NECs to RGCs and from RGCs to IPCs. We next queried a second scRNA-seq dataset from the human developing cortex at GW17 to GW18 (*22*) and found that APP is expressed in all 16 types of cells present in the human developing cortex (Fig. 1, E and F). We also confirmed APP expression in all the regions of human developing cortex from VZ to cortical plate (CP) at GW14 using RNA scope (Fig. 1, G and H). APP mRNA appears enriched on the apical/ventricular pole of RGCs (Fig. 1, I and J). Together, these data show that APP is expressed in human cortical progenitors throughout neurogenesis raising the question of what its function may be at that stage of human cortex development. To gain initial insight into whether APP is potentially required during human fetal development, we queried genetic loss of function data obtained from the human population by the Genome Aggregation Database project (*29*, *30*). These analyses reveal that APP has a loss of function observed/expected (LOEUF) score of 0.42 (https://gnomad.broadinstitute.org/gene/ENSG00000142192?dataset=gnomad_r2_1), meaning that complete loss of APP causes ~60% developmental mortality, a rate unexpectedly higher than that of key neurodevelopmental genes such as Neurog2 (LOEUF = 0.54) and Ascl1 (LOEUF = 0.59) that show less than 50% developmental mortality (Fig. 1K). Together, these data suggest that APP may play an important role in human cortex development.

To test this hypothesis, we generated *APP-KO* iPSCs by CRISPR-Cas9 (fig. S1, A to K; see methods for iPSC lines and experimental details) and chose three *APP-KO* clones and one *APP* isogenic control clone, which received Cas9 but was not mutated, for further analysis. We verified the karyotype and the APP locus in all clones to ensure isogenicity. Upon induction of cortical differentiation, the morphology and molecular identity of neural progenitor cells (NPCs) were confirmed with NESTIN, SOX2, SOX1, and PAX6 (fig. S2). Cortical identity was verified using FOXG1 and EMX2 (fig. S3, A to H). We first confirmed that APP protein is expressed in human cortical progenitors (Fig. 1L). Seven days after initiation of neuronal differentiation, we stained the cells for SOX2 (progenitors), doublecortin (DCX; intermediate progenitors and immature neurons), and Tubulin β-III (TUJ1; neurons) to determine cell type. We observed a notable increase in TUJ1 (fig. S3, I to P) and DCX (fig. S3, Q to X) expression 7 days post differentiation in all three *APP-KO* clones relative to the control clone. To examine whether this effect is general or brain-region specific, we generated motor neurons (*31*) from control and *APP-KO* iPSCs. While human cortical neurogenesis occurs over 3 to 4 months, spinal motor neurons are generated at a much faster rate in 2 to 3 weeks. We used a 17-day protocol with 10 days of neural induction and 7 days of neural differentiation. We chose two early time points (day 1 and day 4 after differentiation; i.e., days 11 and 14 of culture) that approximately correspond to the 7 days postdifferentiation time point in cortical neurons (fig. S4A). We first confirmed the expression of APP protein in motor neuron progenitor cells (day 10 of culture; fig. S4B) and then examined their differentiating daughters for expression of ISLET1 (MN marker) and TUJ1 (fig. S4, C to N). No significant differences were detected in the number of ISLET1^+^ cells in *APP-KO2* and control (fig. S4O) at either time point. These data suggest that loss of *APP* preferentially affects the temporally slower cortical neurogenesis compared to the temporally faster motor neurogenesis.

**Fig. 2.**
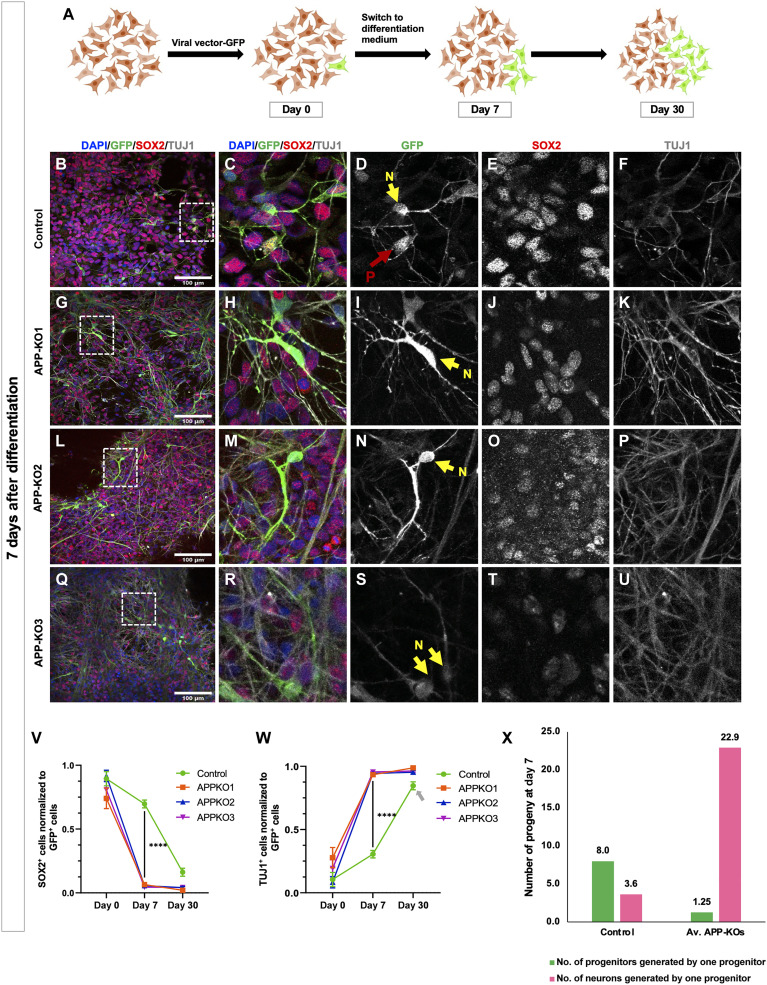
Following neurogenesis using sparse labeling of progenitors confirms increased neurogenesis in *APP-KO* clones. (**A**) Schematic illustration of the sparse labeling approach using a lentiviral vector (*pLv794_pTrip_PromSynapsin1_GFP_DeltaU3)* to follow the fate of the progeny of labelled progenitors (icons adapted from BioRender.com). (**B** to **U**) Representative immunofluorescence images showing expression of SOX2 and TUJ1 within GFP^+^ cells in isogenic control and *APP-KOs* 7 days after differentiation (scale bar, 100 μm; N, neuron; P, progenitor). (**V**) Decrease in the number of SOX2^+^ cells normalized to GFP^+^ cells and (**W**) Increase in the number of TUJ1^+^ cells normalized to GFP^+^ cells in *APP-KO* clones compared to isogenic control within 7 days [two-way analysis of variance (ANOVA), ****P < 0.0001]. At 30 days after differentiation, the ratios of progenitors and neurons have plateaued in *APP-KO* clones. The ratios in isogenic control clone have reached almost similar levels as in KO clones, but they are still on a dropping (progenitors) and rising (neurons, gray arrow) trajectories, respectively. (**X**) Number of progenitors and neurons generated by a single progenitor in control and *APP-KO* at 7 days after differentiation shows that one control NPC produced in average eight NPCs and 3.6 neurons. In contrast, within the same time frame, each APP-KO NPC produced on average 1.25 NPCs and 22.9 neurons. See table S1 for the detail of the numbers. We used six coverslips per condition per time point with the total number of quantified GFP^+^ cells at DAY 0 (Control = 74; *APP-KO1* = 65; *APP-KO2* = 64; *APP-KO3* = 90), day 7 (Control = 814; *APP-KO1* = 860; *APP-KO2* = 1892; *APP-KO3* = 1868) and day 30 (Control = 657; *APP-KO1* = 599; *APP-KO2* = 524; *APP-KO3* = 552).

To determine the neurogenic potential of control and *APP-KO* cortical NPCs, we sparsely labeled them (70 control NPCs and an average of 63.6 NPCs in the 3 *APP-KO* clones; table S1) with *pLv794_pTrip_PromSynapsin1_GFP_DeltaU3*, a green fluorescence protein (GFP)–expressing lenti-viral vector (*32*, *33*) (Fig. 2A) and quantified the number of GFP^+^ cells progenitors (SOX2) and GFP^+^ neurons (TUJ1) at days 0, 7, and 30 after differentiation (Fig. 2, B to U). After 7 days, we observed a significant decrease in NPCs (Fig. 2V) paralleled by a significant increase in neurons over time (Fig. 2W), in all three *APP-KO* compared to control. Notably, while the proportion of neurons generated by *APP-KO* progenitors shows a plateau between days 7 and 30, the slope is still rising in controls (arrow in Fig. 2W), meaning that neurogenesis is still ongoing in the control background, consistent with previous reports (*33*). Quantitatively, we found that one control NPC produced on average eight NPCs and 3.6 neurons after 7 days of differentiation. In contrast, within the same time frame, each *APP-KO* NPC produced on average 1.25 NPCs and 22.9 neurons (Fig. 2X and table S1), indicating a major switch of cortical NPCs from self-amplifying state to neurogenic state. This means that, in the absence of APP, a starting pool of NPCs does not produce more progenitors but is nevertheless maintained to produce neurons.

### Cortical neurons are generated on a shorter time scale in the absence of APP

One of the key features of cortical development is the sequential generation of different neuronal subtypes that will occupy different layers of the neocortex. This feature is preserved in two-dimensional (2D) cultures, whereby early-born (EB; deep layer) neuronal fate markers appear before late-born (LB; upper layer) cell fate markers (*7*, *34*). To assess whether loss of APP affects this order, differentiation was launched, and cells were tracked for 7, 15, and 30 days after differentiation. We first observed clear expression of EB neuronal marker CTIP2 at day 15 followed by SATB2 at day 30 in both genotypes.

**Fig. 3.**
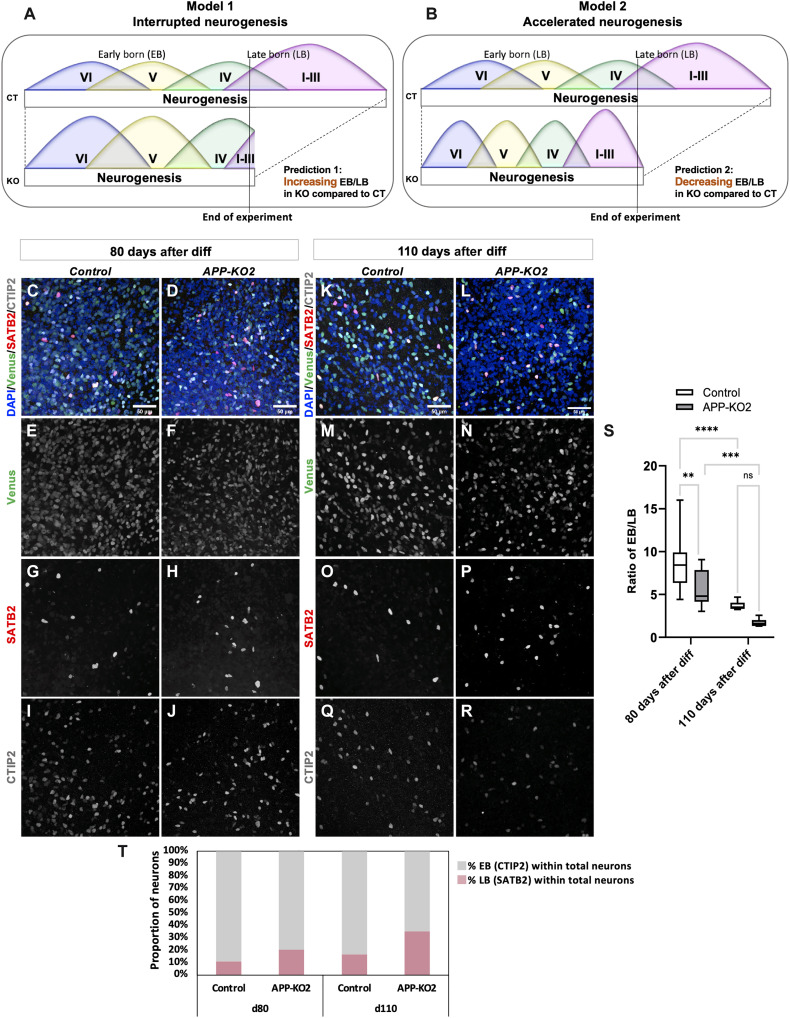
Accelerated production of different types of cortical neuron in *APP-KO* clones. (**A**) The “interrupted neurogenesis” model in which APP KO progenitors are prematurely depleted before producing sufficient LB neurons predicts increasing EB-to-LB neuron ratio compared to isogenic controls (**B**) Conversely, the “accelerated neurogenesis” model in which APP KO progenitors undergo the same temporal neurogenic series but with faster dynamics predicts decreasing EB/LB neuron ratio at the same time point during active neurogenesis. (**C** to **R**) 80 and 110 days postdifferentiation neurons stained for Venus, CTIP2, and SATB2 in isogenic control and *APP-KO2* (scale bar, 50 μm for all the images). (**S**) Lower ratio of EB/LB neurons that would decrease over time in *APP-KO2* compared to isogenic control supports the accelerated neurogenesis model in the absence of APP (*n* = 3, ordinary two-way ANOVA, **P = 0.0025 for 80 days after diff Control versus 80 days after diff *APP-KO2*, ****P < 0.0001 for 80 days versus 110 days after diff Control, ***P = 0.0006 for 80 days versus 110 days after diff APP-KO2). (**T**) Percentage of CTIP2 and SATB2 cells within total neurons at 80 and 110 days after differentiation.

Put together, our data so far can be explained by two very different models. In a first model, which we term the “interrupted neurogenesis” model, *APP-KO* progenitors initiate neurogenesis prematurely causing an exhaustion of the progenitor pool while they are still in their early temporal phases. In a second model, which we term the “accelerated neurogenesis” model, *APP-KO* progenitors go through their entire temporal program but in a much shorter period. These two models make diametrically opposite predictions for the EB-to-LB neuron ratio (EB/LB ratio) at different stage of neurogenesis. The first model predicts that the EB/LB ratio would be higher in *APP-KO* and would increase over time compared to control (Fig. 3A) because the mutant progenitors would be consumed before enough LB neurons are produced. The second model predicts that the EB/LB ratio would be initially lower in *APP-KO* and would decrease over time compared to control (Fig. 3B) because *APP-KO* progenitors would have completed their temporal program while control progenitors are still in early phases of generating LB neurons. However, eventually the control progenitors would “catch up” and the ratio would be similar between the two conditions. To test these predictions, we followed the cultures for 50, 80, and 110 days after differentiation. We first stained 50 days postdifferentiation cultures for the neuronal fate markers FOXP2 (EB) and SATB2 (LB) and found that the ratio of FOXP2/SATB2 (EB/LB ratio) is lower in all three *APP-KO* clones compared to the control reaching significance in two of the three cases (fig. S5, A to N) consistent with the accelerated neurogenesis model. Next, we followed the ratios of neuronal fate at 80 (Fig. 3, C to J) and 110 days (Fig. 3, K to R) after differentiation by transducing progenitors of control and *APP-KO2* with *pLv794_pTrip_PromSynapsin1_Venus-NLS_DeltaU3* and staining their neuronal progeny for Venus, CTIP2 (EB), and SATB2 (LB). We found that the difference in EB/LB ratios (CTIP2/SATB2) within the labeled neurons decreases overtime and is not significant anymore at 110 days after differentiation (Fig. 3, S and T). These data suggest that APP contributes specifically to the absolute temporal scale of human neurogenesis but not the relative temporal birth order of cortical neurons. To have an insight on maturation of control and *APP-KO* cortical neurons, we stained them for synapse markers. At 60 days after differentiation, neither control nor *APP-KO* neurons do express synaptic markers. In contrast, at 120 days after differentiation, both control and *APP-KO* neurons express presynaptic (MUNC13) and postsynaptic (PSD95) markers (fig. S5, O to V), and no significant difference was observed in synapse density per micrometer in control and *APP-KO2 (*fig. S5W). To determine the functional properties of cortical neurons, we produced cortical organoids from control and *APP-KO2* using previously published protocol (*35*), and extracellular recordings were performed from cortical organoids at 160 days of differentiation using the multielectrode array (MEA) system (fig. S6, A and B). Representative raster plots and heat maps of neuronal network activity displays spontaneous activity in both control and *APP-KO2* cortical organoids (fig. S6, C to F). Most organoids displayed spontaneous spiking activity, even though the known variability between organoids was also observed. No significant difference was observed between control and *APP-KO2* for either the number of active electrodes or the mean firing rate (fig. S6, G and H). Together, these data suggest that loss of APP does not significantly alter the density of synapses or functional activity of the neurons.

### Loss of APP drives human cortical progenitors into neurogenic state

**Fig. 4.**
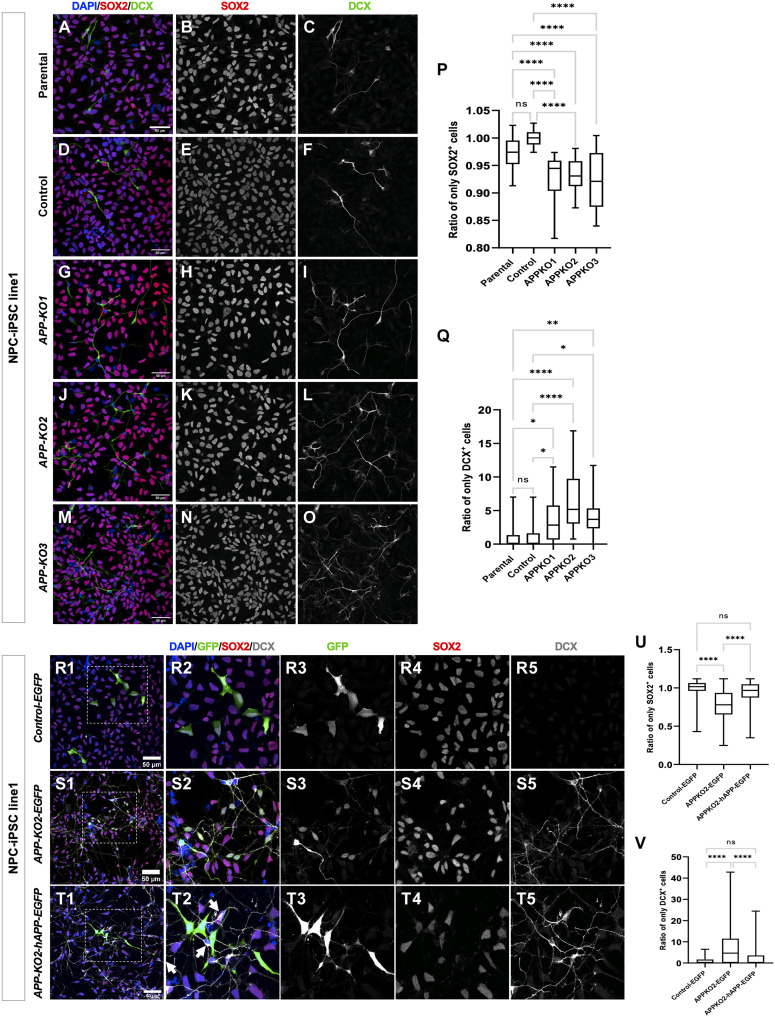
Loss of APP drives premature differentiation of human NPCs. (**A** to **O**) NPCs in proliferating medium from parental, isogenic control and *APP-KOs* stained for SOX2/DCX. (**P**) Significant decrease in ratio of SOX2^+^ cells (*n* = 3, one-way ANOVA, *P* < 0.0001 for all the conditions). (**Q**) Significant increase in ratio of DCX^+^ in *APP-KOs* background compared to parental and isogenic control (*n* = 3, one-way ANOVA, *P = 0.0235, ****P < 0.0001, **P = 0.0061 for parental versus *APP-KO1*, *APP-KO2* and *APP-KO3* respectively. *P = 0.0487, ****P < 0.0001, *P = 0.0151 for isogenic control versus *APP-KO1*, *APP-KO2* and *APP-KO3*, respectively; scale bar, 50 μm). (**R1** to **R5**) Isogenic control NPCs transfected with *pPB-CAG-IRES-EGFP*. (**S1** to **S5**) *APP-KO2* NPCs transfected with *pPB-CAG-IRES-EGFP*. (**T1** to **T5**) *APP-KO2* NPCs transfected with *pPB-CAG-hAPP-IRES-EGFP* and stained for GFP/SOX2/DCX. APP rescued the phenotype of transfected cells but not neighboring cells (arrows in T2). (**U**) Ratio of SOX2^+^ and (**V**) DCX^+^ cells in all three conditions (*n* = 3 independent biological repeat, ordinary one-way ANOVA, ****P < 0.0001; scale bar, 50 μm). We used four coverslips per condition in each biological repeats. In total, 1431, 1252, and 1540 cells were quantified for *Control-EGFP*, *APP-KO2-EGFP*, and *APP-KO2-EGFP-hAPP*, respectively.

Given the expression of APP in fetal human cortical progenitors (*22*, *28*) (Fig. 1, A to J) and the acceleration of neurogenesis observed in *APP-KO* cells, we hypothesized that that APP is required in the progenitors themselves to maintain cortical NPCs in a prolonged self-amplifying progenitor state. To test this idea, we quantified the ratio of NPCs versus IPCs and EB neurons by staining pure NPC cultures for SOX2 and DCX (Fig. 4, A to O) before the onset of neuronal differentiation, where there should be very little spontaneous differentiation under control conditions. Notably, we observed a significant decrease in the ratio of SOX2^+^ cells (Fig. 4P) and a significant increase in the ratio of DCX^+^ cells (Fig. 4Q) in *APP-KO*s relative to the isogenic parental (see Materials and Methods) and guide RNA/Cas9-treated controls. To rule out genetic background effects, we generated an *APP-KO* clone from a different independent iPSC line (iPSC line 2; see Materials and Methods for details) (fig. S1, L to N) and observed the same results in those NPCs (fig. S7, A to K). We also stained cortical organoids from control and *APP-KO* clones of two different iPSC lines for SOX2 and DCX at day 15, when most cells are in the NPC state (iPSC line 1; fig. S7, L to S, and iPSC line2; fig. S7, U to Z″). We performed 3D reconstructions of the organoids and quantified DCX expression (iPSC line 1; movies S1 and S2 and iPSC line2; movies S3 and S4). We observed more total DCX in *APP-KO* organoids relative to control in both genetic backgrounds (fig. S7, T and Z‴).Next, we performed a rescue experiment by transfecting *APP-KO2* NPCs with integrative (PiggyBac) expressing vectors containing either GFP alone (*pPB-CAG-IRES-EGFP*) or GFP and APP (*pPB-CAG-hAPP-IRES-EGFP*) and staining for SOX2 and DCX. Quantification of GFP^+^/SOX2^+^ and GFP^+^/DCX^+^ cells showed that *APP-KO* NPCs that received the APP-containing vector but not GFP alone were restored to a progenitor state and the number of their neuronal progeny was reduced back to control levels (Fig. 4, R1 to V). We noted that restoring APP only rescued NPCs expressing it, but not neighboring cells (arrows in Fig. 4T2), suggesting a cell-autonomous requirement. Together, these data show that APP is cell-autonomously required to repress premature neurogenesis in human cortical NPCs. Last, to test whether this effect on cortical neurogenesis was specific to human cortical NPCs, we tested for signs of premature neuronal differentiation in the *APP-KO* mouse cortex at embryonic day 10.5 (E10.5), a stage when most cells should still be progenitors. Previous studies of the role of APP and its paralogs APLP1 and APLP2 in mouse neurogenesis have consistently reported contradictory results, including increased neurogenesis, decreased neurogenesis, and absence of any effects (*36*–*39*). We examined the developing mouse cortex at E10.5 and observed no Tuj1^+^ cells in the progenitor layer and no difference in Pax6 (NPC) and Tuj1 expression between *App-WT* and *App-KO* brains (fig. S8, A to N). We then examined the number of progenitors (Pax6), EB (Ctip2), and LB (Satb2) neurons at E17.5 toward the end of neurogenesis. We observed no significant change in the number of Satb2-, Ctip2-, or Pax6-positive cells between *App-WT* and *App-KO* brains (fig. S8, O to Z′). These results suggest that APP’s requirement in cortical progenitors may not be as critical in the mouse cortex as it is in humans, perhaps because mouse cortical neurogenesis is much faster.

**Fig. 5.**
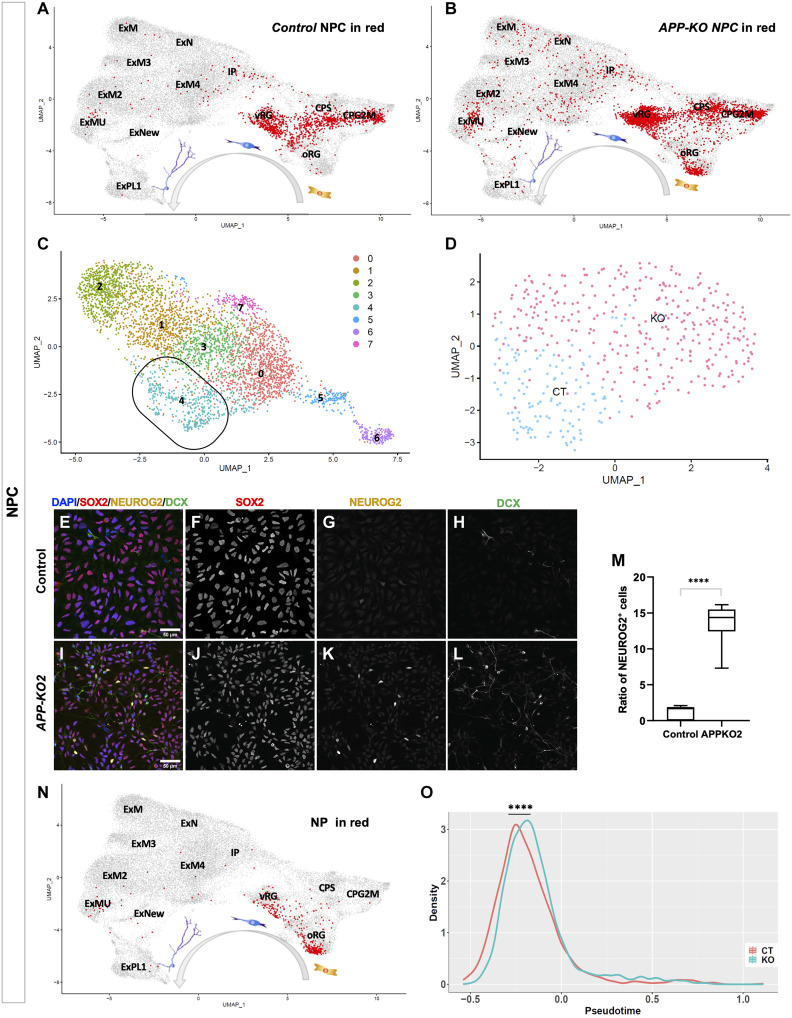
APP-KO NPCs are in a faster temporal trajectory toward neurogenesis. (**A** and **B**) scRNA-seq data from isogenic control and *APP-KO* NPCs both map to normal human progenitor cell types. (**C** and **D**) Cluster analysis without a priori cell identity identified eight clusters most of which are composed of similar ratios of isogenic control and *APP-KO* cells (see fig. S9) with the exception of cluster 4 where the majority of cells in cluster 4 are *APP-KO* NPCs. (**E** to **L**) Staining of isogenic control and *APP-KO2* NPCs for NEUROG2/SOX2/DCX. (**M**) Ratio of NEUROG2^+^ neurogenic NPCs is 15-fold higher in *APP-KO2* compare to isogenic control (unpaired *t* test, *P* < 0.0001; scale bar, 50 μm). (**N**) Cluster 4 cells map to oRG or a transitionary state from vRG to oRG in human fetal progenitors. (**O**) Pseudotime analysis of the scRNA-seq data shows a temporal shift toward a differentiated state in the whole population of *APP-KO* NPCs compared to isogenic control (****P = 8.418 × 10^−13^, Kolmogorov-Smirnov test).

To gain insight into how APP regulates the fate of human cortical NPCs, we performed scRNA-seq on control and *APP-KO* NPCs using a novel, nonmicrofluidics-based method (table S2, BioProject accession number PRJNA678443). First, we confirmed that both control and *APP-KO* NPCs mapped to progenitor cells in the human fetal brain by integrating them with recently reported single-cell transcriptomes (*22*) using Integrated Anchors analysis followed by clustering with Seurat (Fig. 5, A and B) (*40*). Unsupervised clustering identified eight clusters as shown by uniform manifold approximation and projection (UMAP) 2D representation (Fig. 5C). To more precisely identify these cells, we performed clustering analyses according to cell identity, following the cell-type annotation of Polioudakis *et al.* (*22*), which confirmed the presence of closely related control and *APP-KO* clusters (UMAP 2D representation; fig. S9A and heatmaps; fig. S9, B and C) of RGCs, cycling progenitors 1 and 2 (CP1 and CP2), intermediate progenitors (IP) and neurons (N), as well as a cluster that we refer to as the neurogenic progenitors (NP) cluster (see below). NPs represent 13% of the *APP-KO* population and together with the neurons appear at the expense of cycling progenitors, which show a reduction of ~20% in *APP-KO* cells compared to control (fig. S9D), suggesting premature cell cycle exit and increased differentiation of *APP-KO* cells. Consistent with this, we found a 20% reduction in cells expressing the cell cycle marker Ki67 in *APP-KO* NPCs compared to controls (fig. S9, E to G). Because APP is known as stress response gene due to its elevated expression in brain injury and ischemia (*41*), part of this reduction might be due to general loss of cells caused by cell death. We investigated cell death by immunostaining for cleaved caspase 3. Although we found a small but significant increase in cell death in *APP-KO* background compared to control (fig. S9, H to J), the percentage of dying cells was well below 5% and therefore does not explain the observed reduction in proliferation. The NP cluster that represents cluster 4 of the previous analysis (Fig. 5C) contained both control and *APP-KO* cells, with the majority being *APP-KO* (Fig. 5D). To verify the neurogenic state of these progenitors, we performed SOX2/NEUROG2/DCX triple staining (Fig. 5, E to L) on control and *APP-KO* NPCs. NEUROG2 expression marks commitment to differentiation of mammalian NPCs (*42*, *43*). We observed a ~15-fold increase in the number of NEUROG2^+^ progenitors in *APP-KO* compared to control (Fig. 5M). This is close to the increased ratio of neurons to progenitors (~18-fold) we found to be produced by *APP-KO* NPCs (table S1).

To further understand the cell identity [ventricular radial glia (vRG), outer radial glia (oRG), and IPC] of NPs, we mapped them to human fetal brain cells and identified that most of them mapped to oRG or transitionary state from vRG to oRG (Fig. 5N) suggesting APP in the human fetal brain might influence the generation of oRG cells. This is supported by other evidence in our single cell data. First, some of oRG-enriched genes such as *PTPRZ1* and *FABP7* are markers of the NP cluster (table S2). Second, cell type analysis in control and *APP-KO* (fig. S9K) according to Polioudakis *et al.* (*22*), shows that while the majority of progenitors are vRGs under both conditions, there is a an increase in oRGs in APP mutants.

Our scRNA-seq dataset gave us the opportunity to further test the accelerated neurogenesis model by performing pseudotime analysis (*44*, *45*) to determine the temporal trajectory of *APP-KO* NPCs relative to control. Pseudotime analysis shows a significant overall temporal shift toward a differentiated state in the *APP-KO* population compared to control (Fig. 5O) which was true for all classes of progenitors but not IPs and neurons (fig. S9L). It has been reported that lengthening of cell cycle duration increases neurogenesis at the expense of progenitors (*46*–*48*). We used the cumulative labeling assay (*49*) to measure cell cycle length. We treated control and *APP-KO* progenitors with 1 μM Edu and sampling was performed overtime from 2 to 55 hours with Edu continuously in the culture (fig. S10, A to I). While average cell cycle length in control is 36 hours, *APP-KO* progenitors shows protracted cell cycle length about 48 hours (fig. S10J). Together, our data show that loss of *APP* creates an accelerated drive toward neurogenesis in human cortical progenitors, suggesting that APP regulates downstream mechanisms required to maintain cortical NPCs in a progenitor state.

### Loss of APP in human cortical progenitors triggers activator protein–1 activation to drive neurogenesis

**Fig. 6.**
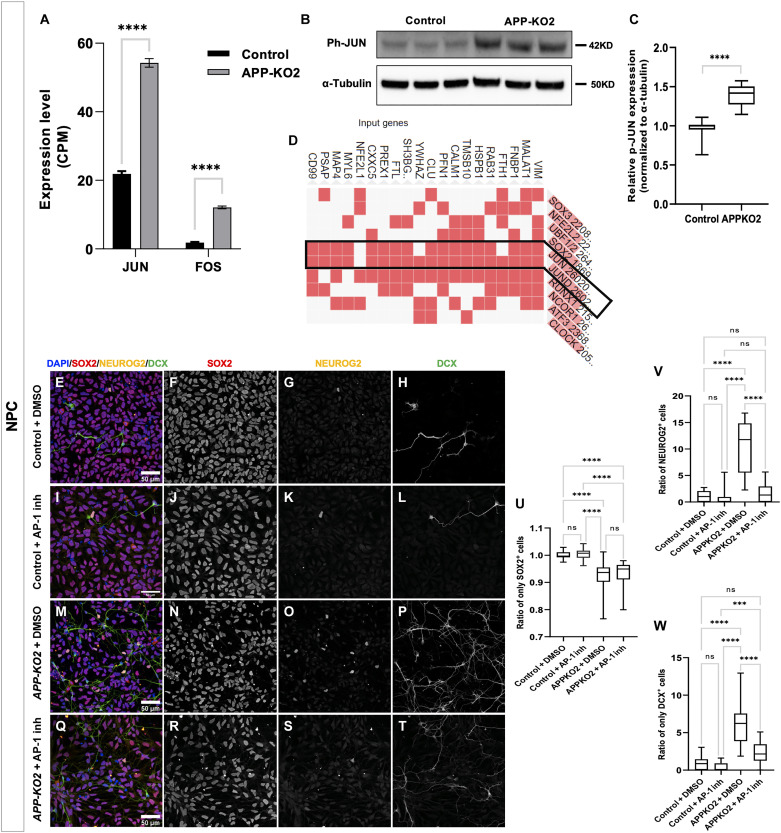
Loss of APP increases differentiation of NPCs through activating the JNK pathway. (**A**) Up-regulation of *JUN* and *FOS* in *APP-KO2* NPCs in bulk RNA sequencing results (ordinary one-way ANOVA, *P* < 0.0001). (**B** and **C**) Western blot shows significant increase in phospho-JUN in *APP-KO2* NPCs compared to isogenic control (*n* = 3 biologically independent repeats, unpaired *t* test, ****P < 0.0001). (**D**) JUN family members bind to marker genes of NPs in the ChEA database (http://amp.pharm.mssm.edu/Enrichr/). (**E** to **T**) NPCs treated with AP-1 inhibitor (AP-1 inh, SR11302) and stained for SOX2/DCX/NEUROG2 showed (**U**) no increase in ratio of SOX2^+^ cells in *APP-KO2* treated with SR11302 compared to untreated *APP-KO2* NPCs (*n* = 3, ordinary one-way ANOVA, ****P < 0.0001 for all the significant condition). (**V**) Significant decrease in NEUROG2^+^ cells in *APP-KO2* treated with SR11302 compared to untreated *APP-KO2* NPCs (*n* = 3, ordinary one-way ANOVA, ****P < 0.0001 for all the significant conditions). (**W**) Significant decrease in DCX^+^ cells in *APP-KO2* treated with SR11302 compared to untreated *APP-KO2* NPCs (*n* = 3, ordinary one-way ANOVA, ****P < 0.0001 for isogenic control-DMSO versus *APP-KO2*-DMSO, isogenic control-SR11302 versus *APP-KO2*-DMSO and *APP-KO2*-DMSO versus *APP-KO2*-SR11302 and ***P = 0.0008 for isogenic control-SR11302 versus *APP-KO2*-SR11302). Scale bar, 50 μm for all the images.

To gain insight into the mechanisms of the gain of neurogenic state in the absence of APP, we performed bulk RNA sequencing in *APP-KO* and control NPCs. We obtained 763 differentially expressed genes (558 up; 205 down including *APP* as expected) between control and *APP-KO2* NPC (table S3 and fig. S11, A and B). Consistent with the *APP-KO* phenotype, bulk RNA-seq showed down-regulation of primary progenitor’s markers *PAX6* and *CDON*, and up-regulation of neurogenic genes *NEUROG2*, *BCL6*, *ASCL1*, *NHLH1*, and *DCX*. Moreover, *CDKN2B*, a well-known cell cycle inhibitor, and differentiation promoting factors *DLL3*, *JAG2*, and *DNER* were also up-regulated (fig. S11C). Consistent with the scRNA-seq data, we observed up-regulation of some oRG markers such as *TNC*, *MOXD1*, *FABP7*, and *ACSBG1* in our bulk data (fig. S11D). Notably, *TNC* expression (4.2 log2FC in *APP-KO* compare to control) was also confirmed by Western blot showing approximately sixfold increase in protein level (fig. S11, E and F). One pathway whose activity is intimately linked to APP function in a variety of organisms and neuronal contexts is the JUN N-terminal kinase (JNK) pathway (*50*) whose effector proteins, JUN and FOS, combine to form the activator protein–1 (AP-1) transcription factor. Significant evidence supports APP-dependent transcriptional repression of c-Jun and reduced basal activity of c-Jun N-terminal kinase (JNK) in PC12 cells (*51*) with activation of the JNK signaling pathway reduced either by APP overexpression or treatment with sAPPα (*51*–*53*). Moreover, *c-Fos* expression is up-regulated in the prefrontal cortex of *App-KO* mice (*54*, *55*). We observed increased expression of *JUN* and *FOS* mRNAs in *APP-KO* NPCs (1.3 and 2.7 log2FC, respectively; Fig. 6A), suggesting that APP also regulates their expression in human context. JUN is activated upon phosphorylation by JNK (*56*–*58*), and we observed a 1.5-fold increase in phospho-JUN in *APP-KO* NPCs compared to control (Fig. 6, B and C). Last, AP-1 regulates the expression of BCL6 in germinal-center B cells (*59*), and BCL6, which we found to be up-regulated in *APP-KO* NPCs, drives neurogenesis of cortical progenitors in the developing mouse cortex (*60*, *61*). Analysis of genes characterizing the *APP-KO* NP cluster of our single-cell transcriptomic dataset showed enrichment for binding of JUN and JUND to their promoters by ChIP-X Enrichment Analysis (ChEA) (*62*); http://amp.pharm.mssm.edu/Enrichr/, Fig. 6D). Furthermore, we also found consensus AP-1 binding sites in the regulatory regions of genes up-regulated in *APP-KO* NPCs in the bulk RNA-seq data, including *DCX* (table S4). To test whether AP-1 activation might drive *APP-KO* NPCs into a neurogenic state, we treated control and *APP-KO2* NPCs with a specific AP-1 inhibitor (SR11302) (*63*, *64*) and stained them for SOX2, NEUROG2, and DCX (Fig. 6, E to T). While the ratio of SOX2^+^ cells remained unchanged after treatment (Fig. 6U), the number of NEUROG2^+^ and DCX^+^ cells was significantly rescued (Fig. 6, V and W). These results suggest that loss of APP causes premature differentiation through AP-1 activation independently of NPC self-renewal, indicating that there is a second APP-dependent mechanism for NPC amplification.

### Loss of APP reduces human cortical progenitor amplification by attenuating the canonical WNT pathway

**Fig. 7.**
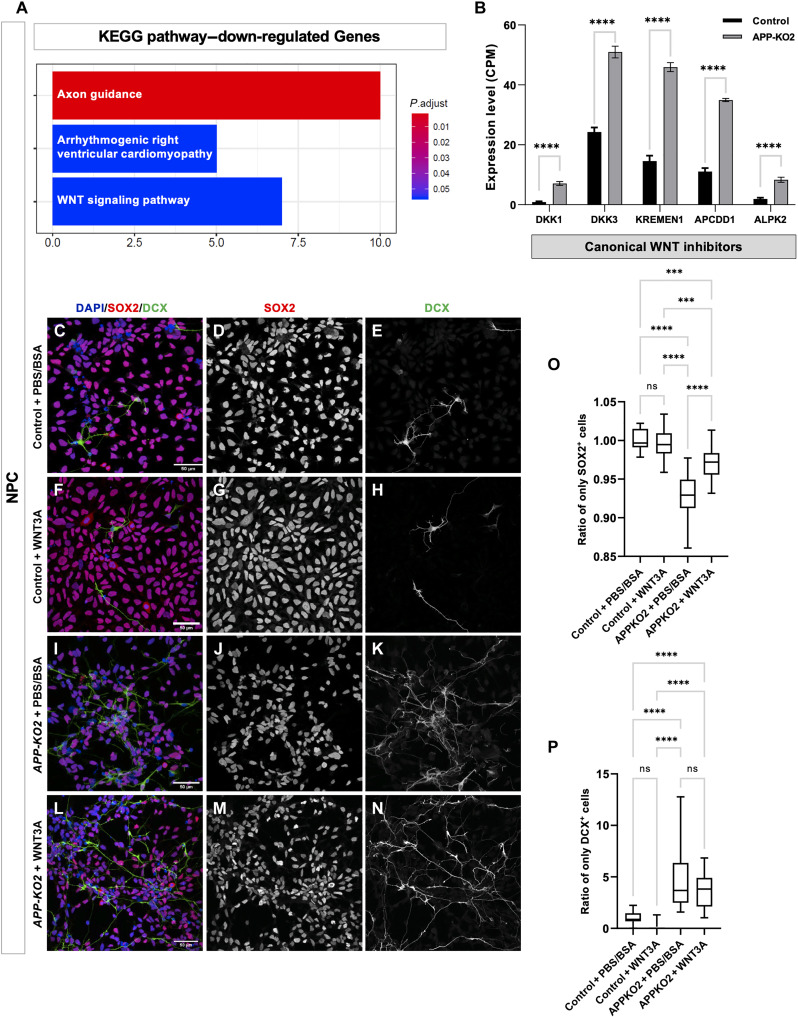
Loss of APP decreases amplification of NPCs by attenuating the canonical WNT pathway. (**A**) KEGG pathway analysis shows down-regulation of WNT signaling pathway in *APP-KO* background. (**B**) Up-regulation of canonical WNT inhibitors *DKK1*, *DKK3*, KREMEN1, *APCDD1*, and *ALPK2* in *APP-KO2* NPC compared to isogenic control (ordinary one-way ANOVA, ****P < 0.0001). (**C** to **N**) Isogenic control and *APP-KO2* NPCs treated with canonical WNT ligand (WNT3A) and stained for SOX2 and DCX. (**O**) Ratio of SOX2^+^ was significantly rescued in *APP-KO2* NPCs treated with WNT3A (*n* = 3, ordinary one-way ANOVA, ****P < 0.0001 for isogenic control + PBS/BSA versus *APP-KO2* + PBS/BSA and *APP-KO2* + PBS/BSA versus *APP-KO2* + WNT3A, ****P = 0.0001 for isogenic control + PBS/BSA versus *APP-KO2* + WNT3A, ***P = 0.0006 for isogenic control + WNT3A versus *APP-KO2* + WNT3a). (**P**) Ratio of DCX^+^ cells was not rescued after treatment with WNT3a (*n* = 3, ordinary one-way ANOVA, ****P < 0.0001 for all the significant conditions).

The canonical WNT contain different proteins including Frizzled family of proteins as main receptors, co-receptors LRP5/6 and downstream protein β-catenin and is required in a variety of contexts for stem cell maintenance (*65*, *66*). Kyoto Encyclopedia of Genes and Genomes (KEGG) pathway analysis of down-regulated genes in our bulk RNA-seq data indicated that WNT signaling is reduced in *APP-KO* NPCs (Fig. 7A), with up-regulation of canonical WNT inhibitors *DKK1*, *DKK3*, *KREMEN1*, *APCDD1*, and *ALPK2* (Fig. 7B). We also observed down-regulation of WNT7A and up-regulation of WNT7B in our dataset. We asked whether this reduction in WNT signaling might contribute to reduced NPC amplification in *APP-KO* NPCs. WNT3A is a prototypical example of a ligand that consistently activates the canonical WNT pathway (*21*, *22*). We compared control and *APP-KO* NPCs treated with the canonical ligand WNT3A for 48 hours to untreated NPCs and stained them for SOX2 and DCX (Fig. 7, C to N) and proliferation marker Ki67^+^ (fig. S12, A to H). As expected, Wnt3A treatment induced increased proliferation in control progenitors. In APP-KO progenitors, we observed a complete rescue in proliferation (fig. S12I), a partial but significant rescue of the loss of SOX2^+^, but no reduction in DCX^+^ cells (Fig. 7, O and P). Thus, consistent with all our analyses, APP-KO progenitors lose their progenitor status more readily even when they are induced to proliferate but require suppression of JNK signaling to differentiate.

## DISCUSSION

Here, we report what is, to our knowledge, the first evidence for a genetic network that specifically regulates the absolute timing at which human cortical progenitors generate neurons. Our evidence suggests that this timing requires simultaneously sufficient levels of canonical WNT signaling to promote progenitor identity and proliferation as well as sufficient repression of AP-1 activation to inhibit neurogenesis. We find that these two processes are genetically coupled by the activity of the APP and can be uncoupled downstream of it. Our evidence supports a model in which this genetic network is required for the prolonged maintenance of human cortical NPCs in a progenitor state. In this model, loss of APP reduces WNT signaling and activates AP-1 thus lowering the threshold for NPCs to transition from a self-renewing progenitor state to a neurogenic state. Notably, NPCs lacking APP generate cortical neurons in the correct temporal order. This means that the absolute amount of time a human cortical progenitor takes to generate neurons can be genetically uncoupled from the relative temporal order of the generation of cortical neurons. Obviously, evolution has achieved such uncoupling in the timing of cortical development in different mammalian species, where the same subtypes of neurons are generated in the same relative temporal sequence despite massive differences in the total amount of time required for cortical neurogenesis. Our findings suggest either that loss of APP mimics this evolutionary uncoupling or, more excitingly, that regulation of genes in the APP–WNT–AP-1 network may be part of the evolutionary uncoupling mechanism. Last, it is noteworthy that the acceleration of neurogenesis upon loss of APP does not appear to alter the final ratio of EB to LB neurons and does not appear to result in significantly faster maturation of the neurons produced. This contrasts with accelerated neuronal production using cocktails of small-molecule inhibitors, including the Notch signaling inhibitor DAPT, which appears to accelerate both processes (*67*). This may indicate the clock of neuronal production and the clock of neuronal maturation are genetically separable and that APP is specifically required for the former. Nevertheless, several points remain to be clarified in future work. First, it will be important to determine whether the acceleration is proportional along the entire duration of cortical neurogenesis or whether the effect varies over time, especially during the generation of upper layer neurons. Second, it will be interesting to determine whether the neuronal maturation clock is completely normal or whether there are also subtle but critical, effects yet to be found, such as changes in axonal branching and/or dendritic spine complexity, as we have recently observed in mouse cortical neurons (*68*). Last, determining whether prematurely born APP-KO human cortical neurons can maintain a healthy differentiated state over time or whether they have a shorter life span will be very interesting to investigate.

We observed an increase in the percentage and molecular markers of outer/basal RGCs upon loss of APP. Outer/basal radial glia are a progenitor population that was massively increased during the evolution of the primate brain. Together with an increase in NPs, this may suggest that the APP-dependent temporal program regulates the proportion of different types of progenitors in the developing human cortex. An indirect consequence of this notion would be that the relative spatial organization of the VZ and outer SVZ of the human fetal cortex is, in part, a derivative of the temporal progression of progenitor fate. In addition, whether the greater requirement for APP in humans compared to rodents may offer a clue into fAD is worth further investigation. We noted the up-regulation of *MAPT* gene (1.36 log2FC, table S3), encoding the other key AD protein Tau, in *APP* mutant progenitors. It is tempting to speculate that fAD mutations in *APP* have subtle effects on early human cortical development that cause reduced cortical robustness and resistance to a lifetime of neuronal and glial stress partially contributing to premature neurodegeneration.

Three observations suggest that human brain development may be particularly sensitive to loss of APP. First, there is a strongly reduced tolerance to complete loss of APP in human genetic data (*29*, *69*–*71*). Second, both in vivo and in vitro studies on mouse NPCs lacking APP have shown no consistent evidence for altered neurogenesis (*8*, *36*, *37*). Third, among the genes whose expression is altered in *APP-KO* NPCs, 33 have been reported to be located within human accelerated regions, which are conserved genomic loci with elevated divergence in humans (*72*, *73*). We also did not observe any effect on human motor neurogenesis, suggesting that APP function may be more important during cortex development. Because APP is highly conserved in sequence and expression among mammalian species, we speculate that the notable requirement for APP in human cortical neurogenesis is less due to anything special about the human APP protein itself and more due to the special features of human cortical neurogenesis. In particular, we propose that the links between APP, WNT, and JNK pathways have been co-opted in human, and perhaps more generally primates, to prolong the ability of the cortical NPCs to retain a progenitor state while also generating more differentiated daughters. For example, human/primate cortical NPCs may express human/primate-specific regulators of APP activity or stability that allow them to suppress JNK signaling and enhance WNT signaling more efficiently than in other mammalian species.

We have recently shown that APP is a co-receptor for Wnt-3a and that Wnt-3a binding protects APP from degradation (*68*). We observed that WNT receptor FZD5 that mediates both canonical and noncanonical pathway (*74*, *75*) is up-regulated (2.4 L2Fc) in our bulk RNA-seq data. It is possible that the rescue of progenitor proliferation defects observed in *APP-KO* NPCs by WNT3A is partially mediated by up-regulation of FZD5. Furthermore, it has recently been reported that there is a correlation between down-regulation of WNT signaling and emergence of oRG cells meaning cortical organoids generated using Triple-i (Dual-SMAD and WNT inhibition) specifically enrich for oRG cells compare to classical Dual-SMAD inhibition protocol (*76*). This is in line with our findings that WNT signaling pathway is down-regulated in APP-KO progenitors and *APP-KO* progenitors show a shift toward oRG fate. Together, these data suggest that APP regulates the balance between NPC proliferation and differentiation through partially parallel mechanisms, facilitating WNT signaling to promote NPC proliferation and repressing AP-1 levels to prevent premature NPC differentiation. In addition, human cortical NPCs may be particularly sensitive to cellular stress. Some studies suggest that increased stress drives premature neurogenesis in cortical progenitors (*77*). APP is a conserved stress response protein, as are JUN and FOS. Therefore, the absence of APP may mimic cellular stress, activate AP-1–mediated responses, and drive premature neurogenesis. We have previously shown that the coding sequence contributes to evolutionary changes in the neurogenic activity of proneural proteins (*78*). We cannot exclude that subtle changes to the otherwise highly conserved APP coding sequence may be involved in its acquisition of this function in human cortical NPCs.

Increasing evidence points to human-specific genetic alterations, such as new gene isoforms (*33*, *79*–*82*), microRNAs (*83*), and regulatory sequences, creating human-specific transcriptional networks (*72*, *84*) potentially resulting in human-specific brain features. A tantalizing speculation is that members of the genetic network we uncovered here may be regulated by one or more human-specific protein isoform or microRNA whose expression is enriched in the cortex. A clinical case of a homozygous nonsense truncating mutation in APP leading to microcephaly has been reported (*70*, *71*). The reported patient carries mutations in three genes including homozygous mutations in *APP*, that strongly reduce its expression levels, and *SETX* as well as a heterozygous mutation in *CLN8*. Because *CLN8* is associated with autosomal-recessive neuronal ceroid lipofuscinosis [Mendelian Inheritance in Man (MIM): 600143] and the patient is heterozygote for that, the authors conclude that the phenotype is not related to this gene. *SETX* is associated with autosomal-dominant juvenile amyotrophic lateral sclerosis type 4 (ALS; MIM: 602433) and autosomal recessive ataxia with ocular apraxia type 2 (AOA2; MIM: 606002). Because ALS and AOA2 usually have onset in adolescence, they conclude that it is more probable that the phenotype is related to the mutation of *APP* and not *SETX*. This supports the notion that human brain development is vulnerable to loss of APP and that perhaps the status of *APP* heterozygosity should be included in clinical genetic counseling.

## MATERIALS AND METHODS

### iPSC culture and maintenance

iPSC cell lines WTSIi002-A (https://cells.ebisc.org/WTSIi002-A/, iPSC line1) and WTSIi008-A (https://cells.ebisc.org/WTSIi008-A/, iPSC line2) were purchased from EBISC (European Bank for Induced pluripotent Stem Cells) and maintained on Geltrex LDEV-Free hESC-qualified Reduced Growth Factor Basement Membrane Matrix (Thermo Fisher Scientific) in Essential 8 Flex Media Kit (Thermo Fisher Scientific) with 0.1% penicillin/streptomycin. Cultures were fed every other day and passaged every 5 to 7 days by ReLeSR (STEMCELL Technologies).

### Creating *APP-KO* clones by CRISPR-Cas9

#### iPSC line1

Four guide RNAs were designed by Crispor (http://crispor.tefor.net/) to target APP’s first exon and cloned in pCAG-CAS9-GFP (fig. S1, A and B). To evaluate the cleavage efficiencies of the guide RNAs, human embryonic kidney–293 cells were transfected with p-CAG-Cas9-GFP containing four guide RNAs (fig. S1C). DNA extraction and polymerase chain reaction (PCR) were performed 48 hours after transfection for the cleaved region. Sequenced PCR products were analyzed in TIDE (Tracking of Indels by Decomposition) to obtain cleavage efficiency. The sequence of each guide RNA, their cleavage efficiency, and off-target are listed in table S5. The best guide RNA with 8.5% total efficiency and lowest off-target was chosen for further experiments (fig. S1D). Karyotyping was performed on the iPSC line (which we refer to as “parental line” 1) before the CRISPR approach and the results showed a deletion in chromosome 18. The iPSC line was transfected with the best guide RNA using Lipofectamine stem reagent (Thermo Fisher Scientific). GFP-positive cells were isolated by fluorescence-activated cell sorting 48 hours after transfection and seeded at low density. A total of 118 clones were picked after 5 days and expanded, and 60 were screened for possible mutations (fig. S1, E to H). Sixteen of the 60 clones had homozygous mutations for APP. These mutations are classified as 1-, 2-, 3-, 20-, 22-, and 33-bp deletions and 1- and 2-bp insertions (table S6). The absence of expression of the APP protein was confirmed by Western blot in 14 clones (fig. S1I). The efficiency of producing APP knock-out clones was 23% (fig. S1J). Three APP knock-out clones were chosen for further experiments, and low APP mRNA levels were confirmed by quantitative PCR (fig. S1K). The control, which was chosen for further experiments, expressed guide RNA and CAS9, but the APP gene remained un-mutated. Karyotyping was performed for the *APP-KO* clones, control, and parental iPSC. The results showed that the same deletion in chromosome 18 in parental line was also confirmed in control and *APP-KO* clones. No other deletion or duplication was observed in *APP-KO* clones and control after CRISPR approach. The pluripotency of *APP-KO* cells and the control were confirmed by octamer binding transcription factor 4 (OCT4) staining (fig. S2, D to G).

#### iPSC line2

Karyotyping was performed on the iPSC line before the CRISPR approach (which we refer to as “parental line” 2), and the results showed a duplication in chromosome 20. The guide RNA 1 and the ribonucleoprotein approach were used to generate *APP-KO*. A total of 1 × 10^6^ iPSCs were nucleofected with the ribonucleoprotein complex from Integrated DNA Technologies (225 pmol of each RNA and 120 pmol of Cas9 protein). iPSCs were plated 48 hours later at very low density (10 cells/cm^2^) on Laminin-521 with CloneR supplement (Stem Cell Technology) for clonal selection. One week later, iPSC clones were picked under a stereomicroscope and cultured on Laminin-521 in 96 well plates (Duscher). The resulting iPSC clones were duplicated after confluency and used for cryoconservation and DNA extraction. One base-pair deletion was observed in screening results and the absence of expression of APP protein was confirmed by Western blot. The APP knock-out clone and control were used for further experiments, and the low level of APP mRNA was confirmed by quantitative PCR (fig. S1, L to N). Karyotyping was also performed for the *APP-KO* clone, control, and parental iPSC. The results confirmed the same duplication in chromosome 22 in all the conditions. No other deletion or duplication was observed in *APP-KO* clones and control after CRISPR approach.

### Cortical differentiation of human iPSC

The cortical differentiation protocol is based on Dual-SMAD inhibition (*85*, *86*) with some modification. The protocol is divided into two main steps: neural induction and neural differentiation. At day 0, iPSCs were detached by Accutase (Thermo Fisher Scientific) and transferred to T75 ultralow attachment flasks (VWR) in Essential 8 medium with 0.1% penicillin/streptomycin and Stemgent hES Cell Cloning & Recovery Supplement (Ozyme, 01-0014-500) to form embryoid body. At day 1, the medium was switched to embryoid body medium containing Dulbecco’s modified Eagle’s medium/F12 Glutamax, 20% knockout serum replacement, 1% nonessential amino acids, 1% penicillin/streptomycin, and 0.55 mM 2-mercaptoethanol and supplemented with dorsomorphin (1 μM, Sigma-Aldrich, P5499), SB431542 (10 μM, Abcam, ab120163) for 8 days. At day 8, embryoid bodies were collected and seeded on Geltrex and maintained for 6 to 8 days in Neurobasal without vitamin A medium and B27 without vitamin A supplemented with human epidermal growth factor (EGF; 10 ng/ml) and human fibroblast growth factor 2 (FGF2) (10 ng/ml). Cells were fed every day from day 0 to day 16. Rosettes were manually picked at day 15 and dissociated with Accutase, seeded on poly-ornithine and laminin-coated dishes for expansion, and maintained with passage for two additional weeks to achieve a large pool of NPCs (fig. S2, A to C). Characterization for NPCs at passage numbers 3 to 5 was performed by staining for Nestin, SOX2, SOX1, and PAX6 (fig. S2, H1 to K6) and FOXG1 and EMX2 (fig. S3, A to H). NPC with passage numbers 4 to 9 was used for the experiments. We used control and APP-KO NPC with similar passage numbers in all experiment to avoid unknown effects of passage number. NPCs were seeded on 24-well plates with coverslips, and the day after seeding, the medium was switched to the B-27 Plus Neuronal Culture System (Thermo Fisher Scientific) supplemented with ascorbic acid. Cortical neurons were stained for EB neural markers and LB neural markers at the indicated early time points or kept for 3 to 4 months in culture and stained for synaptic markers at 120 days after differentiation.

### Labeling and tracing of NPCs for short-term neuronal culture

Sparse labeling was performed using viral vector approach as described (*32*, *33*). We used GFP-expressing lent viral vector (*pLv794_pTrip_PromSynapsin1_GFP_DeltaU3*). We tested the vector before performing any experiment with different concentration of virus [MOI (multiplicity of infection) = 1, MOI = 0.1, MOI = 0.05, and MOI = 0.01]. First, we found that the *pLv794_pTrip_PromSynapsin1_GFP_DeltaU3* promoter does initiate GFP expression in SOX2^+^ progenitors and is thus an appropriate tool. Second, we found that MOI = 1 and MOI = 0.5 concentrations were toxic and thus chose MOI = 0.05 for the experiment. Next, APP-KO and control NPCs were transduced by *pLv794_pTrip_PromSynapsin1_GFP_DeltaU3*. The medium was changed to fresh Neurobasal without vitamin A and B27 without vitamin A supplemented with EGF and hFGF2 to wash out lentiviruses 1 day after initial infection. When the GFP^+^ cells appeared (4 days after transduction), it was considered day 0 and the medium was switched to differentiation medium (B-27 Plus Neuronal Culture System) and kept for 7 and 30 days. Lentiviral-expressing cells were detected by anti-GFP antibody, and cell type was determined by anti-SOX2 and anti-TUJ1 antibodies.

### Labeling and tracing of NPCs for long-term neuronal culture

*APP-KO* and control NPCs were seeded on poly-l-ornithine/laminin-coated coverslips and transduced by lentiviral vector containing Venus-NLS (*pLv794_pTrip_PromSynapsin1_Venus-NLS_DeltaU3*). When the Venus^+^ cells appeared, it was considered as day 0 and the medium was switched to differentiation medium (B-27 Plus Neuronal Culture System) and kept for 80 and 110 days. Venus expressing cells were detected by anti-GFP antibody and EB and LB neurons were detected by the antibodies listed in table S7.

### Motor neuron differentiation and staining

Control and mutant iPSC clones were differentiated into motor neurons as described (*31*). In summary, after dissociation of embryoid bodies containing motor neuron progenitors at D10, single cells were plated on poly ornithine-laminin–coated coverslips at 2 × 10^5^ cells per coverslip for 1, 4, and 7 more days. Cells were fixed with 4% paraformaldehyde (PFA) in phosphate-buffered saline (PBS) for 10 min at room temperature (RT). For staining, cells were treated with 5% goat serum with 0.1% Triton X-100 (Thermo Fisher Scientific) for 1 hour at RT and then incubated overnight at 4°C with a mouse anti–βIII-Tubulin (TUJ1, Sigma-Aldrich, 1/500) and a rabbit anti-ISLET1 (Abcam, 1/100) antibodies in 5% goat serum. Secondary antibodies [goat anti-mouse IgG2a-Alexa 555 (1/2000) and goat anti-rabbit IgG Alexa 488 (1/2000) from Thermo Fisher Scientific] were incubated for 1 hour at RT with Hoechst33342 to stain nuclei.

### Immunocytochemistry

Cells were fixed in 4% PFA and incubated for 30 min in blocking buffer (PBS and 0.3% Triton X-100 and 5% horse serum). Primary antibodies (table S7) were diluted in antibody solution (PBS and 0.1% Triton X-100 and 1% horse serum) and applied overnight at RT. After three washes in PBS, secondary antibodies conjugated to Alexa fluorophores (Molecular Probes, Eugene, OR, USA) were diluted at 1:1000 in antibody solution and applied for 1 hour at RT. Cells were washed in PBS followed by nuclear staining by DAPI (Sigma-Aldrich) for 10 min. Cells were washed three more times and mounted by VECTASHIELD Mounting Medium-Vector Laboratories. Confocal image acquisition was performed using Olympus FV1200 and SP8 Leica DLS.

### Generation of cortical organoids

Cortical organoids were generated from human iPSCs using a previously reported protocol (*35*), with some modifications. Human iPSCs were incubated with Accutase (Life Technologies, A1110501) at 37°C for 7 min and dissociated into single cells. To obtain uniformly sized spheroids, approximately 3 × 10^6^ single cells were added per well in the AggreWell 800 plate (STEMCELL Technologies, 34815) with Essential 8 flex medium supplemented with Stemgent hES Cell Cloning & Recovery Supplement (1X, Ozyme, STE01-0014-500) and incubated at 37°C with 5% CO_2_. After 24 hours, spheroids from each microwell were collected by firmly pipetting medium in the well up and down and transferred into Corning nontreated culture dishes (Merck, CLS430591-500EA) in TeSR-E6 (StemCell Technologies, no. 05946) supplemented with two inhibitors of the SMAD signaling pathway, dorsomorphin (2.5 μM, Sigma-Aldrich, P5499) and SB-431542 (10 μM, Abcam, ab120163). From day 2 to day 5, TeSR-E6 supplemented with dorsomorphin and SB-431542 was changed every day. On the sixth day in suspension, neural spheroids were transferred to neural medium containing Neurobasal minus vitamin A (Life Technologies, 10888), B-27 supplement without vitamin A (Life Technologies, 12587), GlutaMax (1%, Life Technologies, 35050), Penicillin-Streptomycin (1%, Life technologies, 15140122) and 2-mercaptoethanol (5 mM, Life Technologies, 31350010). The neural medium was supplemented with EGF (10 ng/ml; PreproTech, AF-100-15) and basic FGF (10 ng/ml; R&D Systems, 234-FSE-025) and changed daily until day 12. From day 12 to day 24, medium was changed every other day. At day 25, the medium was switched to the B-27 Plus Neuronal Culture System (Thermo Fisher Scientific) supplemented with ascorbic acid. Cortical organoids were fed twice per week and kept for 3 to 4 months in culture. They were stained at day 15 for SOX2 and DCX.

### Organoid collection, immunohistochemistry, and imaging

#### 3D staining

At day 15, organoids were fixed with 4% PFA at 4°C for 6 hours and rinsed three times with PBS for 10 min. Following two washing with PBS + 2% Triton X-100 for 2 hours, spheroids were treated overnight at RT with PBS + 2% Triton X-100 + 2% Tween 20 + 20% dimethyl sulfoxide. Blocking/permeabilization was performed with PBS + 10% horse serum, 3% bovine serum albumin (BSA), and 2% Triton X-100 for 24 hours at RT. Primary antibodies (Sox2 1/500, Millipore AB5603 and DCX 1/2000, Millipore AB2253) were incubated in the same solution supplemented with 0.05% Azide for 3 days at 4°C. After multiple washing with PBS + 0.5% Tween 20 until the next day, organoids were then incubated for 24 hours with secondary antibodies. After 24 hours of washing with PBS + 0.5% Tween 20, samples were cleared in RapiClear 1.49 (SunJin lab) before mounting on cavity slides (Hecht Karl 42410010) and confocal image acquisition was performed using Nikon A1R HD25.

#### DCX quantification

Whole-mount day 15 organoids were imaged with a Nikon A1R HD25 confocal microscope at 1-μm intervals with a 10× objective (MRD71120). Analyses on organoids were performed using the NIS-Elements software (Nikon) and Imaris. Image stacks were reconstructed in 3D to measure volume of DCX clusters and total 4′,6-diamino-2-phenylindol (DAPI). Each measurement was normalized to the total volume of DAPI in the organoid. Each organoid is considered as *n* = 1.

### Quantification of synapse markers

The quantification was performed using a semiautomatic method using two plugins in imageJ. First, Neurite tracing was drawn manually by NeuronJ plugin (www.imagescience.org/meijering/software/neuronj/) followed by quantification of synapses by SynapCountJ plugin (*87*).

### MEA recording on organoids

Spike activity was recorded using a 64-channel MEA system (MED64, Alpha MED Scientific). Cortical organoids at day 160 were collected 48 to 72 hours after changing the culture medium and recordings were performed in this conditioned culture media. The organoids were carefully placed into a poly-l-ornithine/laminin–coated MEA well (MED-P515A) and immobilized by a small platinum anchor. Recordings started after a period of recovery of 2 min. Extracellular field potentials were filtered with a band-pass filter (100 Hz and 10 kHz as cutoff frequencies) and acquired at a sampling rate of 20 kHz. Recordings were performed at 37°C. Spikes were detected using MOBIUS software with a detection threshold set at six times the SDs of the estimated noise for each electrode. Data were analyzed using a home-made R script. Electrodes that detected at least 5 spikes per minute were classified as active electrodes.

### RNA scope on human fetal brain samples

Formalin-fixed paraffin-embedded fetal brain sections at 14 weeks of gestation were obtained from the brain collection “Hôpitaux Universitaires de l’Est Parisien – Neuropathologie du développement” (Biobank identification number BB-0033-00082). For all cases studied, informed consent was obtained for autopsy of the brain and histological examination. After removal, brains were fixed with formalin for 5 to 12 weeks. Macroscopic analysis was performed allowing the selection and conditioning of samples (paraffin embedding, 7-μm slicing) of brain tissue for histological analysis. The RNAScope assay was performed according to the standard provider’s protocol (ACD Bio-Techne). In summary, slides were deparaffinized twice with xylene for 5 min, followed by ethanol treatment for three times, and dried at 60°C for 30 min. Peroxidase blocking with H_2_O_2_ performed at RT for 10 min. After washing with water, antigen retrieval was performed in a pressure cooker, followed by rinsing with water and ethanol for 3 min. Slides were air-dried for 10 min and baked at 60°C for 30 min, followed by rinsing with PBS. TrueView (Vector Laboratories, SP-8400-15) applied according to the provider’s recommendations to reduce sample autofluorescence, followed by three times washing with PBS. Protease Plus treatment was applied for 30 min. RNAscope Probe-Hs-APP (C1) (Bio-Techne 418321) hybridization was performed for 2.5 hours, followed by rinsing twice with washing buffer. Slides were kept ON in 4× Saline-Sodium Citrate and amplification steps were performed the day after. The fluorophore OPAL 650 used to label the APP probe. Sections were stained with DAPI (1:5000) and sealed with VECTASHIELD Vibrance Mounting Medium (Vector Laboratories H-1700) and allowed to polymerize O.N. in the dark. Confocal image acquisition was performed using SP8 Leica DLS.

### Embryo collection, immunohistochemistry, and antibodies

E10.5 and E17.5 embryos were collected from *App-WT* or *App-KO* pregnant mice, whole embryos were fixed in 2% PFA in PBS at 4°C for 2 to 3 hours, then dehydrated in 30% sucrose in 1× PBS overnight (o/n). After all the samples sank into the bottom of the tube, they were embedded in OCT compound (TissueTek) and frozen at −20°C. Sagittal sections were performed by cryostat (Leica) at 20 μm and then slices stored at −80°C. For the immunostaining, sections were fixed with 4% PFA for 10 min at RT, then blocked with 10% normal donkey or goat serum in 1× PBS with 0.1% TritonX-100 (PBT) for 1 hour at RT followed by three washes in 1× PBT. Thereafter, these sections were incubated with primary antibodies diluted in 0.1% 1× PBT containing 1% normal donkey or goat serum o/n at 4°C or 3 to 4 hours at RT. After three washes with 1× PBT, samples were incubated with appropriate secondary antibodies conjugated with Alexa Fluor 488, Alexa Fluor 555, or Alexa Fluor 647 (1:500, Invitrogen) in 0.1% 1× PBT containing 1% normal donkey or goat serum for 1 to 2 hours at RT. After washing with 1× PBT three times and then counterstained with DAPI (1:2000, Sigma-Aldrich), the slides were mounted by using VECTASHIELD (Vector) after rinsing. After staining, images were obtained by using a confocal microscope, Olympus FV-1200.

### Quantitative PCR

The cells were lysed directly in the wells by addition of 300 μl of Buffer RLT supplemented with 15 mM β-mercaptoethanol (Thermo Fisher Scientific) after a wash with Dulbecco’s PBS (DPBS, Life Technologies). Total RNA was isolated using the RNeasy Mini extraction kit (Qiagen, Courtaboeuf, France) according to the manufacturer’s protocol. RNA levels and quality were quantified using a NanoDrop spectrophotometer. cDNA synthesis was performed by Thermo Fisher Scientific Verso cDNA Synthesis Kit and Quantitative PCR assay was performed by Sybergreen Gene Expression Assays in triplicate wells of 96-well plates. Primers are listed in table S8 and 2^−(Cp GOI−Cp internal gene)^ was used for analysis. The housekeeping gene (glyceraldehyde-3-phosphate dehydrogenase) was selected to control for variation in cDNA amounts.

### Bulk RNA sequencing and analysis

The cells were lysed directly in the wells by addition of 300 μl of Buffer RLT supplemented with 15 mM beta-mercaptoethanol (Thermo Fisher Scientific) after a wash with DPBS (Life Technologies). Total RNA was isolated using the RNeasy Mini extraction kit (Qiagen, Courtaboeuf, France) according to the manufacturer’s protocol. RNA levels and quality were quantified using a NanoDrop spectrophotometer. RNA sample purity/integrity was assessed using an Agilent 2200 Tapestation. mRNA library preparation was completed following the manufacturer’s recommendations (KAPA mRNA hyperprep ROCHE). Final samples pooled library prep were sequenced on NextSeq 500 ILLUMINA with MidOutPut cartridge (2 × 130 million 75 base reads) with one run, corresponding to 2 × 20 million reads per sample after demultiplexing. The quality of raw data was evaluated with FastQC. Poor-quality sequences were trimmed or removed with fastp software to retain only good quality paired reads without adapters. Star v2.5.3a (*88*) was used to align reads on the hg19 reference genome using standard options. Quantification of gene and isoform abundances was carried out with rsem 1.2.28 (*89*), before normalization on library size with the edgeR (*90*) bioconductor package. Last, differential analysis was conducted with the glm framework likelihood ratio test from edgeR. Multiple hypotheses adjusted *P* values were calculated with the Benjamini-Hochberg (*91*) procedure to control FDR.

### scRNA-seq and analysis

For each control and knockout cell line, single cell samples were prepared for 3′ mRNA sequence determinations using the Scipio bioscience protocol (*92*) with barcoding beads (NxSeq Single-cell RNA-seq Beads, LGC Biosearch Technologies). For beads that captured mRNA molecules, reverse transcription, PCR amplification of cDNA, and sequencing library preparation were performed according to published procedures (*93*). A total of 35,106,546 reads, 56,108,512 reads, and 54,702,467 reads were generated by NovaSeq from two replicates of *APP-KO2* (KO2-1 and KO2-2) and one CT (control) sample, corresponding to 2000, 2000, and 1500 cells. The reads passed QC process using FASTQC v0.11.8. Sample analyses were performed using UMI-tools v1.0.0. Reads were aligned to GRCh38.94 using STAR v2.7 with default parameters. FeatureCounts v1.6.4 (*94*) (Ensembl GRCh38.94.GTF) was used to count the number of aligned reads per feature, followed by umi_tools dedup to collapse those reads belonging to a barcode and mapping the same position of a gene. To build the count table umi_tools count was applied. Seurat V3.1.4 (*40*) was used: (i) to filter cells having more than 200 genes and less than 20% mitochondrial genes, with 1326, 1594, and 1299 cells passing the QC filters for the KO2-1, KO2-2, and CT samples, respectively.; (ii) to pool both KO samples (2920 total); (iii) to cluster KO and CT cells using Seurat clustering with 10 PCA dimensions and resolution 0.5; (iv) to identify cluster markers using FindAllMarkers with default parameters. Visualizations were performed with Seurat and ggplot2 packages. DEGs in KO versus CT were analyzed for enrichment in gene ontology, biological and disease associations using Enrichr tools to further explore ChIP-seq datasets linking transcription regulators with DEGs (*95*). Integration with other early fetal cells (*22*) was performed using Integrated Anchors analysis followed by clustering with Seurat (*40*).

### Pseudo time analysis

The pseudotime was performed on the basis of a machine learning ordinal regression algorithm (*44*, *45*), using the annotated stages of differentiation present in the UMAP 1 component (CP1 > RGC > CP2 > IP > N), ordered from the most undifferentiated to most differentiated ones. Using the control cells (CT), the model adapts weights (that can be considered as importance) to genes so that when using the expression of the genes and their corresponding weights, the algorithm orders the cells along the differentiation axis to build a pseudotime axis, and the genes weights was used to predict the pseudotime distribution of the *APP-KO* cells (KO).

### Rescue experiments

NPCs of Control and *APP-KO2* were transfected with *pPB-CAG-IRES-EGFP* and *APP-KO2* NPCs were transfected by *pPB-CAG-hAPP- IRES -EGFP* and kept for 4 days. Cells were fixed and stained for GFP/SOX2/DCX. The GFP^+^ cells were quantified, and cell type was determined by SOX2 and DCX as progenitor and differentiated cell, respectively.

### Cell cycle length measurement

For measuring cell-cycle length, 1 μM EdU was added to the NPC culture medium. After 2, 4, 14, 24, 30, 36, 48, and 55 further hours in culture, cells were fixed with 4% PFA, and EdU incorporation was visualized using the Click-iT imaging kit (Life Technologies). Cell-cycle lengths were calculated from cumulative labeling as described (*49*).

### Neural precursor treatment

Recombinant human WNT3A (R&D Systems) and SR11302 (TOCRIS) were used at final concentrations of 150 ng/ml and 10 μM, respectively. NPCs of control and *APP-KO2* were treated for 48 hours and then stained for SOX2 and DCX.

### Western blot

Cells were placed on ice, lysed directly in wells by adding RIPA buffer (Sigma-Aldrich) supplemented with Complete protease inhibitor cocktail (Roche), and agitated for 20 min. Thereafter, the samples were collected and centrifuged at >14000 g, 30 min at 4°C and the supernatant was transferred to a fresh tube. Protein determination was performed using the Pierce Detergent Compatible Bradford Assay Kit (Thermo Fisher Scientific). Sample buffer (BOLT LDS, Life technologies) was added to equal amounts of protein, and samples were loaded onto a BOLT 4 to 12% Bis-tris gel and transferred using MES buffer (all from Life technologies). Proteins were blotted onto a 0.2 μM nitrocellulose membrane (GE Healthcare) using semi-dry technique. Membranes were blocked in 5% Nonfat-Dried Milk bovine (Sigma-Aldrich) and incubated overnight at 4°C with primary antibodies listed in table S7. After washing, membranes were incubated with horseradish peroxidase–conjugated secondary antibodies for 1 hour at RT. Protein detection was performed by Pierce ECL Western Blotting substrate (Thermo Fisher Scientific) and bands were visualized using ChemiDoc Touch Imaging System (BioRad Laboratories). Band intensities were calculated using Image Lab software.

### Statistical analysis

Statistical analysis was performed by GraphPad Prism. Data in figure panels reflect three independent experiments performed on different days. An estimate of variation within each group of data is indicated using SEM. We performed unpaired *t* test for assessing the significance of differences in the analyses containing two conditions, one-way analysis of variance (ANOVA) correction in the analyses containing more than three conditions and two-way ANOVA in the group analysis.

## Supplementary Materials

### This PDF file includes:

Figs. S1 to S12 Tables S1, S4 to S8 Legends for tables S2 and S3 Legends for movies S1 to S4

### Other Supplementary Material for this manuscript includes the following:

Movies S1 to S4

Lentiviral vector sequence fasta file

## Notes

View/request a protocol for this paper from *Bio-protocol*.
