# Supplementary material for: The temporal balance between self-renewal and differentiation of human neural stem cells requires the amyloid precursor protein
Source: Sci Adv. 2023 Jun 16;9(24):eadd5002. doi: 10.1126/sciadv.add5002 (PMC10275593; doi:10.1126/sciadv.add5002)
Supplement: Supplementary file 1 — Figs. S1 to S12 Tables S1, S4 to S8 Legends for tables S2 and S3 Legends for movies S1 to S4 [file sciadv.add5002_sm.pdf]

Science Advances
AAAS

Supplementary Materials for

The temporal balance between self-renewal and differentiation of human neural stem cells requires the amyloid precursor protein

Khadijeh Shabani *et al.*

Corresponding author: Bassem A. Hassan, bassem.hassan@icm-institute.org

Sci. Adv. **9**, eadd5002 (2023)
DOI: 10.1126/sciadv.add5002

The PDF file includes:

Figs. S1 to S12
Tables S1, S4 to S8
Legends for tables S2 and S3
Legends for movies S1 to S4

Other Supplementary Material for this manuscript includes the following:

Tables S2 and S3
Movies S1 to S4
Lentiviral vector sequence fasta file

Shabani-Fig.S1

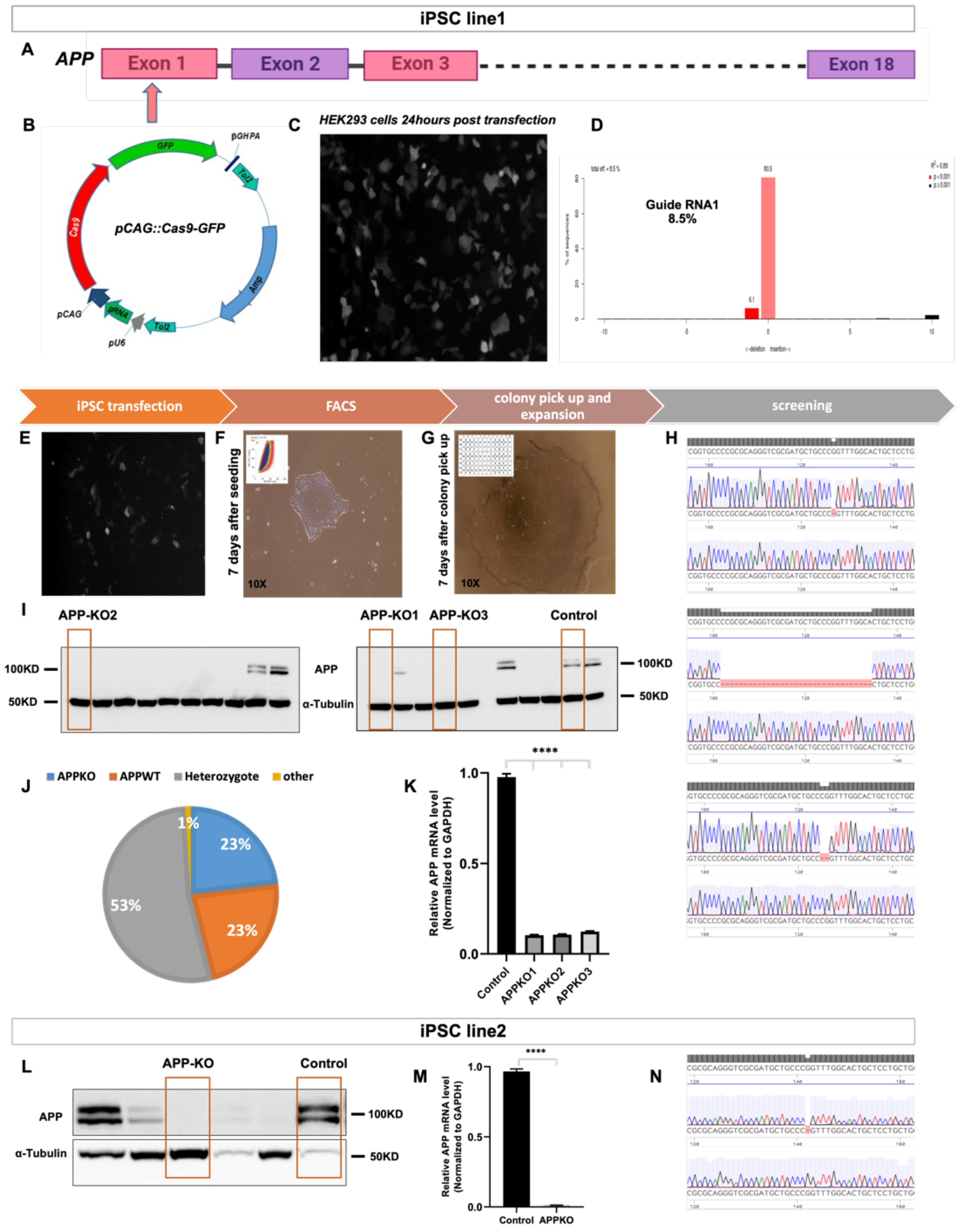

Fig. S1. Generation of iPSC derived APP knockout clones. **(A-K)** Individual steps in generating *APP* knock out from iPSC line WTSIi002-A, henceforth “Line 1”. (**A**) Targeting exon1 of the *APP* gene by **(B)** a plasmid vector containing the guide RNA, CAS9 and GFP. (**C**) HEK293 cells 24 hours after transfection. (**D**) Cleavage efficiency of Guide RNA1 by TIDE (Tracking of Indels by Decomposition) that was used for transfection of iPSC line 1. (**E-H**) iPSC transfection by Guide RNA1, post FACS morphology and sequencing results of clones. (**I**) Western blot results showing undetectable APP protein in 14 out of 60 clones. One isogenic control transfected with Cas9 and the guide RNAi but not mutated for APP and three *APP-KO* clones were chosen for further experiments. (**J**) Distribution of clones with different *APP* genotypes (23%=*APP-KO*, 23%=*APPWT* and 53%=heterozygote and 1%=other mutation). (**K**) qPCR confirms low level of *APP* mRNA expression in *APP-KO* clones (p<0.0001). (**L-N**) Generation of *APP-KO* clones from iPSC line WTSIi008-A, henceforth “Line 2”, confirming very low level of APP mRNA and undetectable APP protein (p<0.0001).

Shabani-Fig.S2

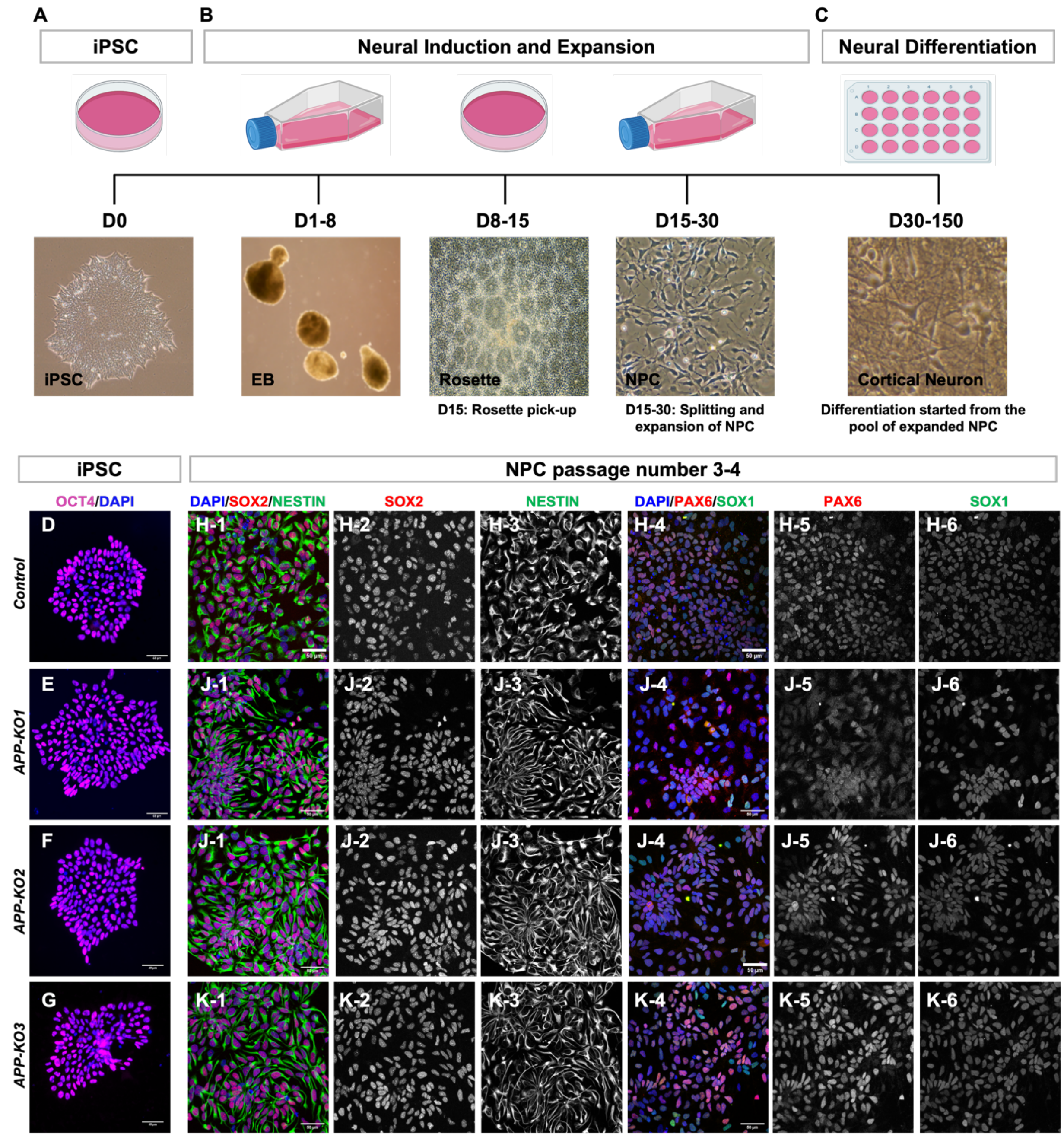

Fig. S2. Timeline of the cortical differentiation protocol and characterization of neural progenitor cells. (A-C) Schematic showing different steps in generating cortical neurons (for more detail see Methods; culture tools adapted from icons by BioRender.com). (**D-G)** Confirming pluripotency of isogenic control and *APP-KO* iPSCs with pluripotency marker OCT4. (**H1-K6**) Characterization of isogenic control and *APP-KO* derived neural progenitor cells at passage number 3-4 with markers: Nestin, SOX2, PAX6, SOX1 (scale bar 50μm).

Shabani-Fig.S3

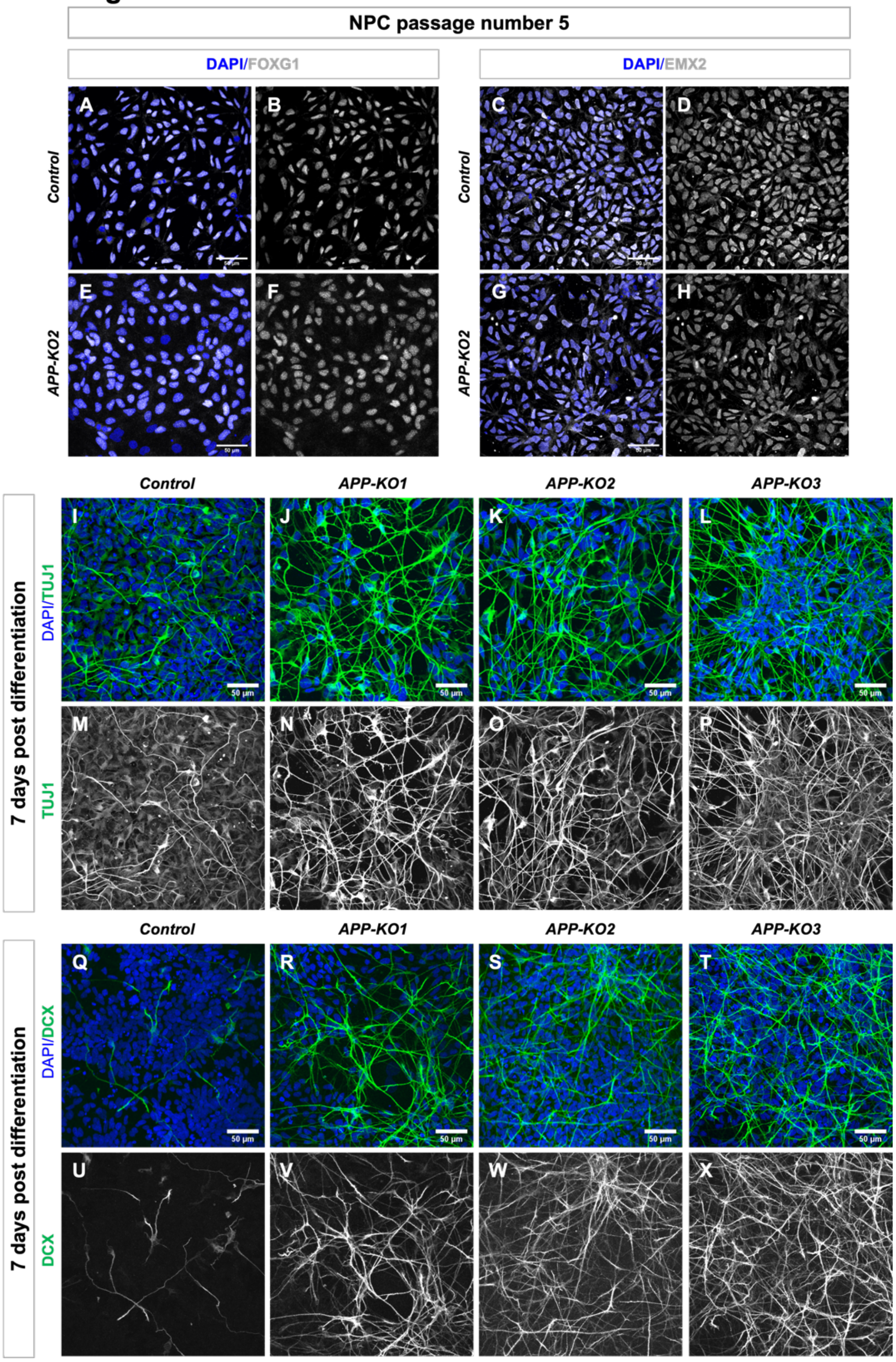

Fig. S3. Further characterization of NPCs and Increased production of young neurons in *APP-KO* background 7 days after onset of neuronal differentiation. (A-H) Characterization of isogenic control and *APP-KO2* derived neural progenitor cells at passage number 5 with markers FOXG1 and EMX2. **(I-P)** Many more cells express the neuronal markers TUJ1 in all *APP-KOs* compared to isogenic control 7 days post differentiation. **(Q-X)** An increase in the number of cells expressing the newly born neuron marker Doublecortin (DCX) in *APP-KO* background compared to isogenic control 7 days post-differentiation (Scale bar 50 µm for all the images).

Shabani-Fig.S4

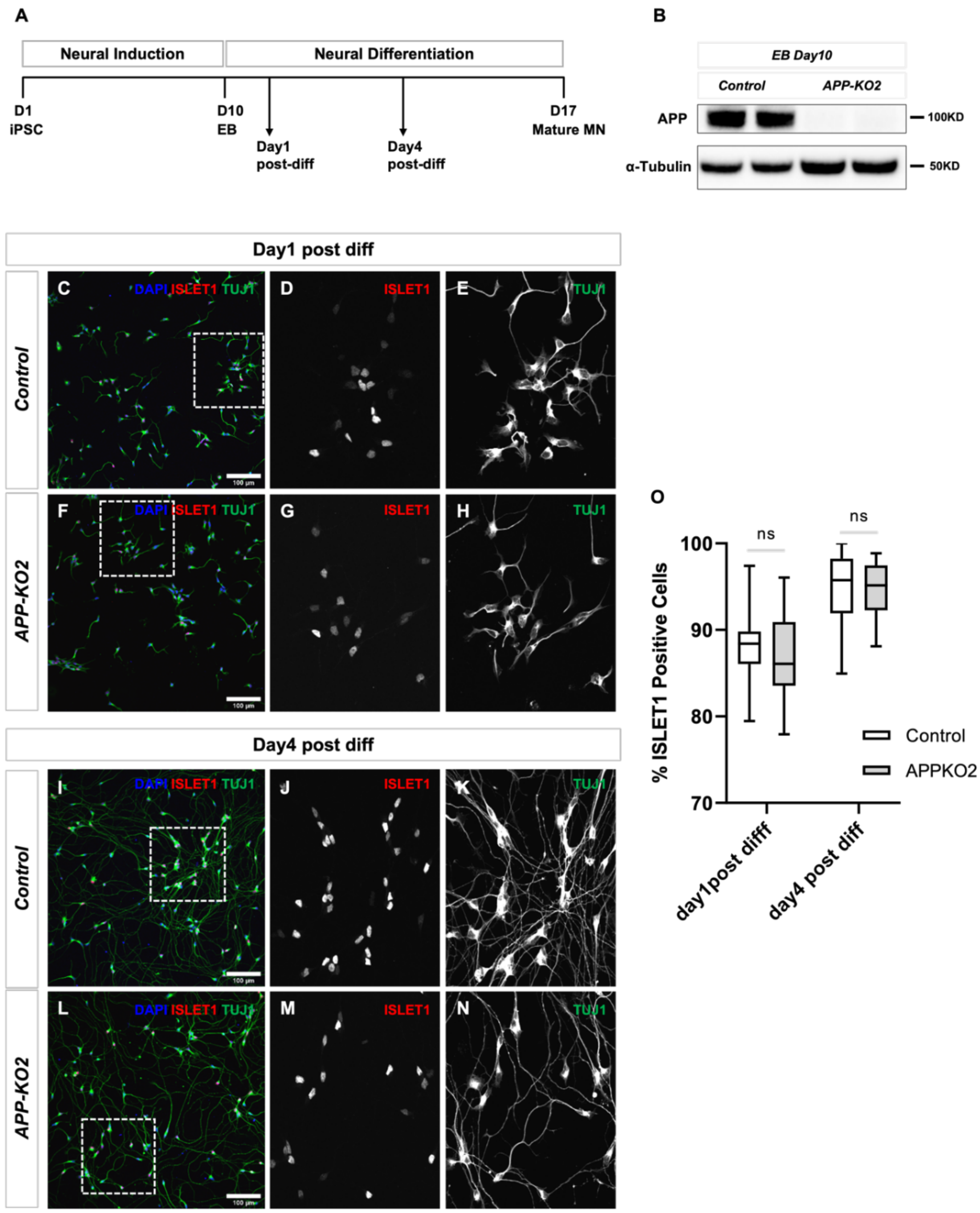

Fig. S4. No difference in the number of ISLET1+ motor neurons derived from isogenic control and *APP-KO2*. **(A)** Schematic showing the timeline of different steps in generating motor neurons. **(B)** APP expression in EB day 10 -contain motor neuron precursor cells- was confirmed in isogenic control motor neuron progenitors by western blot. **(C-H)** Day1 and **(I-N)** Day4 post-differentiation motor neurons stained for ISLET1 and TUJ1 (scale bar 100µm). **(O)** No significant differences were observed in the percentage of ISLET1+ cells at 2 different time points, day1 and day4 post-differentiation (n=3 biological independent repeat, 2way ANOVA). We used two coverslips per condition in each biological repeats. In total, 2914 and 2703 cells at day 1 and 2604 and 2620 cells at day 4 were quantified for control and *APP-KO2*, respectively.

Shabani-Fig.S5

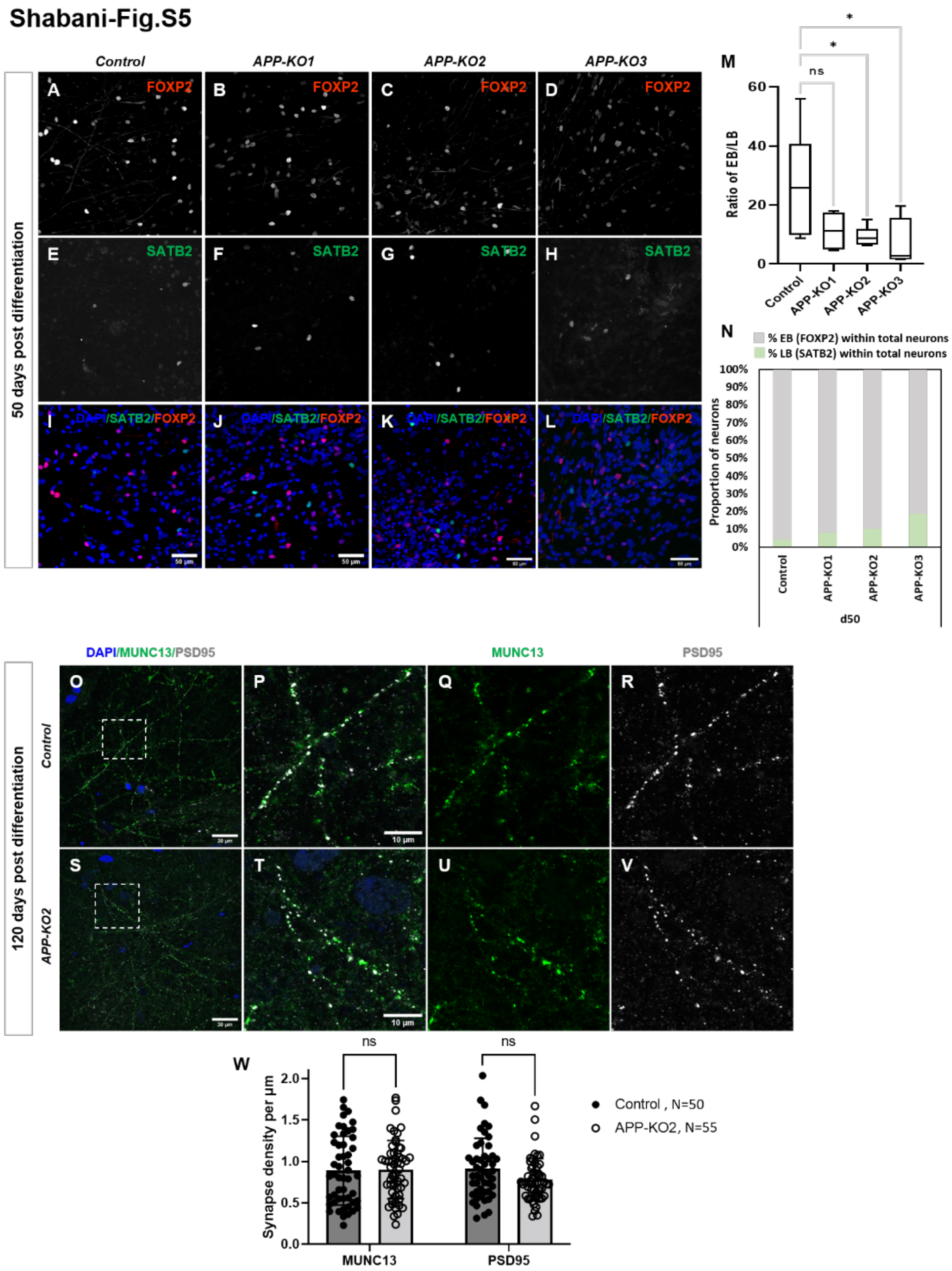

Fig. S5. Staining for cortical neuron markers at 50 days post differentiation and synapse marker at 120 days post differentiation. (A-L) 50 days post differentiation neurons stained for FOXP2 and SATB2 in isogenic control and *APP-KOs* (scale bar 50µm). **(M)** Lower ratio of EB/LB neurons in *APP-KOs* compared to isogenic control supports the accelerated neurogenesis model in the absence of APP (n=1, Ordinary one-way ANOVA, p=0.0557 for isogenic control vs *APP-KO1*, p=0.0373 for isogenic control vs *APP-KO2*, p=0.0252 for isogenic control vs *APP-KO3*). **(N)** Percentage of FOXP2 and SATB2 cells within total neurons at 50 days post differentiation. **(O-V)** Cortical neurons at day 120 post differentiation stained for synapse markers; presynaptic (MUNC13) and post-synaptic (PSD95) (scale bars 30 and10µm). **(W)** No significant difference was observed in the synapse density per µm in control and *APP-KO2*. Number of counted branch (N) is 50 in control and 55 in *APP-KO2.*

Shabani-Fig.S6

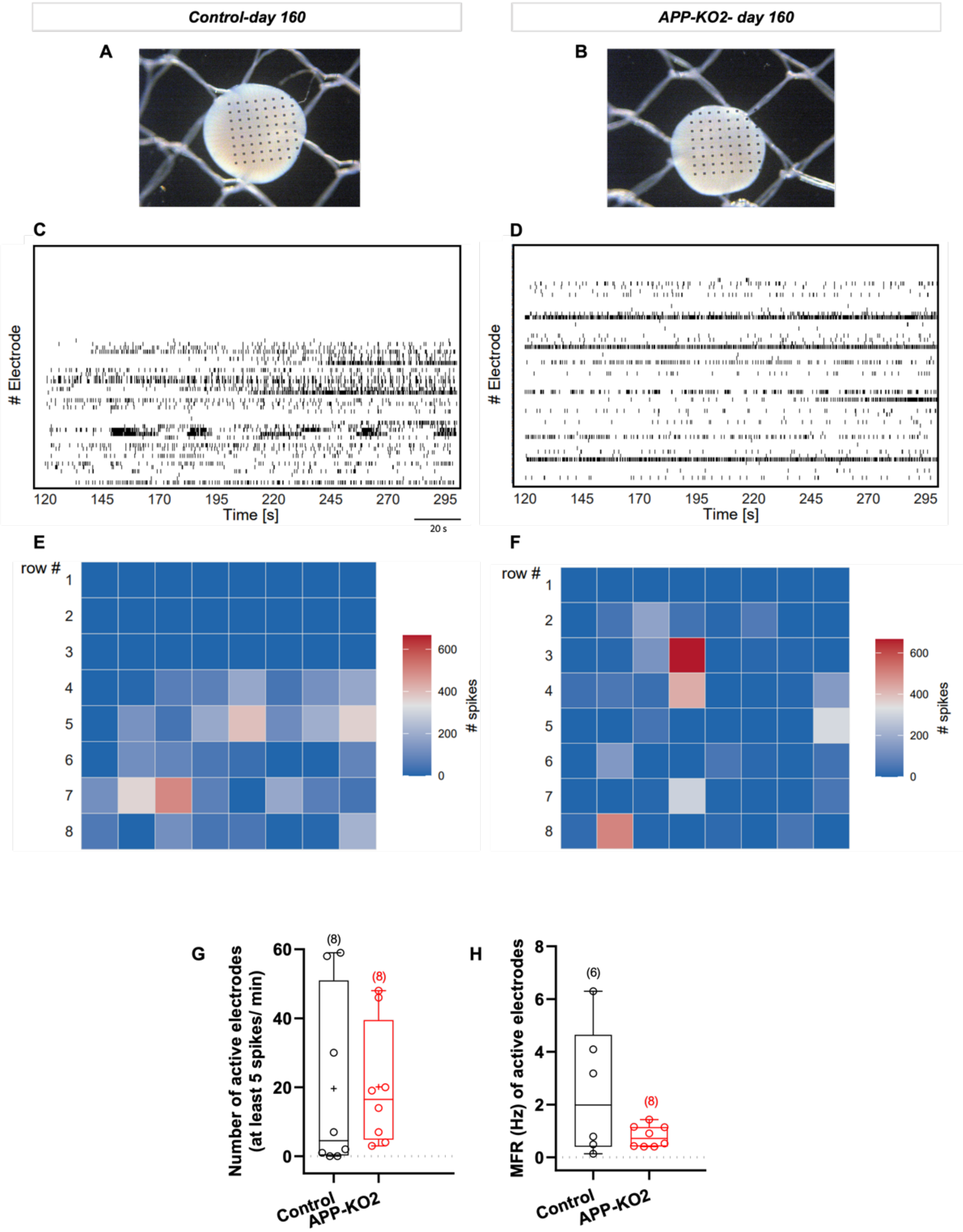

Fig. S6. Functional characterization of cortical organoids at day 160 using MEA recordings reveals no significant difference in *APP-KO2* organoids neuronal network activity compared to control. (A-B) Bright field image of a cortical organoid plated on a 64-electrode MEA. **(C-F)** Representative raster plots and heat maps of control and *APP-KO2* cortical organoids neuronal network activity. Heat map shows spike number in a 180s time frame. Cortical organoids display spontaneous activity at 160 days of their culture **(G-H)** Quantification of number of active electrodes and mean firing rate (MFR) in control and *APP-KO2* ($p=0.442$ and $p=0.414$, respectively). Statistical analyses were performed using Mann–Whitney U test. Data are presented as box plot with median and quartile value. Every dot represents spiking activity of a 3 min recordings of a single organoid (n = 8 from 2 batches).

Shabani-Fig.S7

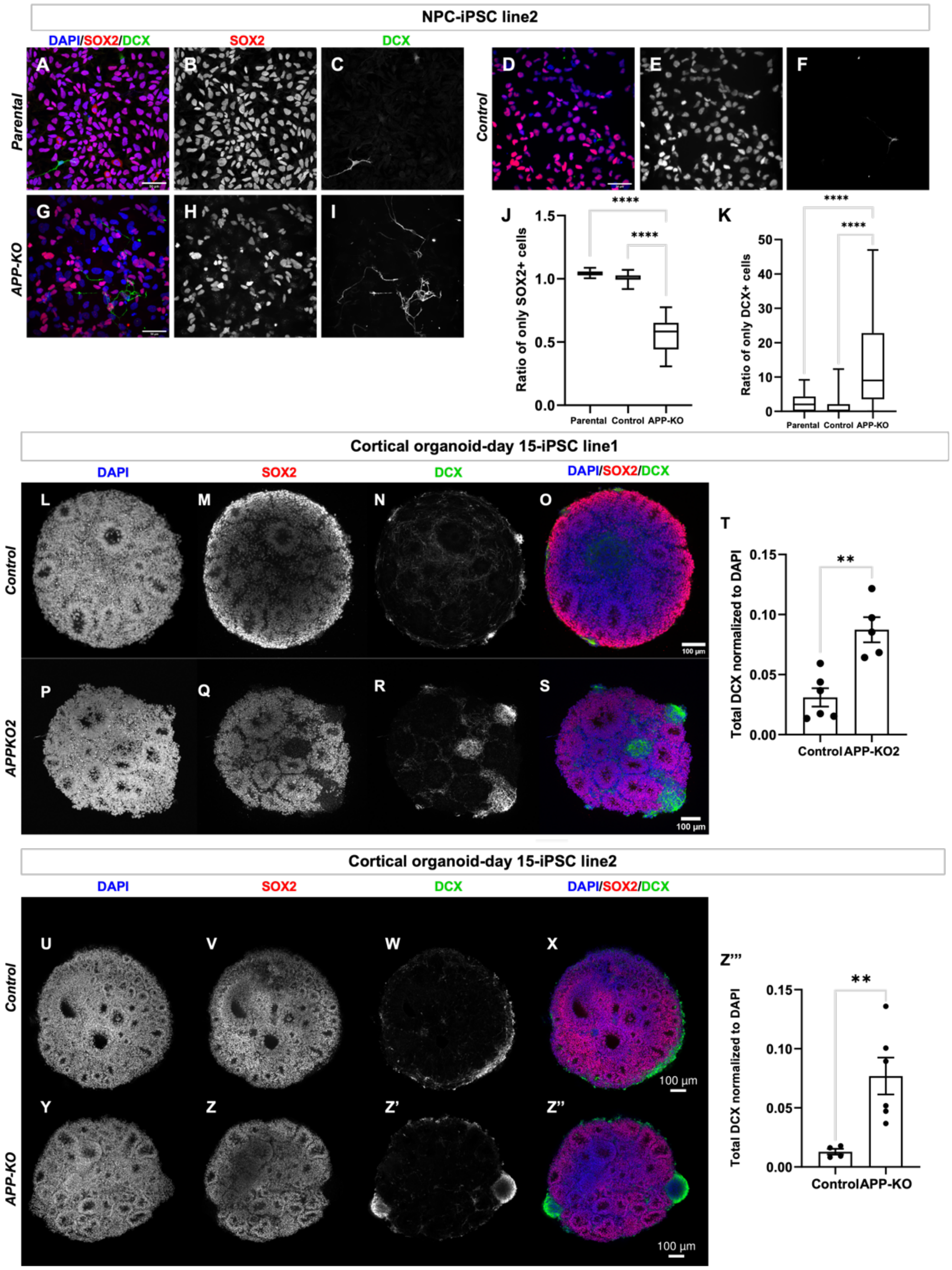

Fig. S7. Premature differentiation due to loss of APP is reproducible in NPC and cortical organoid from different genetic background. (A-I) NPCs from parental, isogenic control (i.e. transfected with guide RNA and Cas9 but not mutated for APP) and *APP-KO* from iPSC line 2 stained for SOX2/DCX. (**J)** Quantification of SOX2+ cells and (**K)** DCX+ in NPCs showed that the results were reproducible independent from genetic background (n=3, Ordinary one-way ANOVA, p<0.0001, scale bar 50µm). Cortical organoid for isogenic control and *APP-KO2* from iPSC line 1 (**L-S)** and iPSC line 2 **(U-Z'')** stained for SOX2/DCX at day 15 of culture showing **(T)** significant increase in total DCX in iPSC1 (p=0.0016, n=6 organoids for isogenic control and n=5 organoids for *APP-KO2*, scale bar 100µm, supplementary videos I-II) and **(Z''')** iPSC2 (p=0.0089, n=4 organoids for isogenic control and n=6 organoids for *APP-KO*, scale bar 100µm, supplementary movies III-IV).

Shabani-Fig.S8

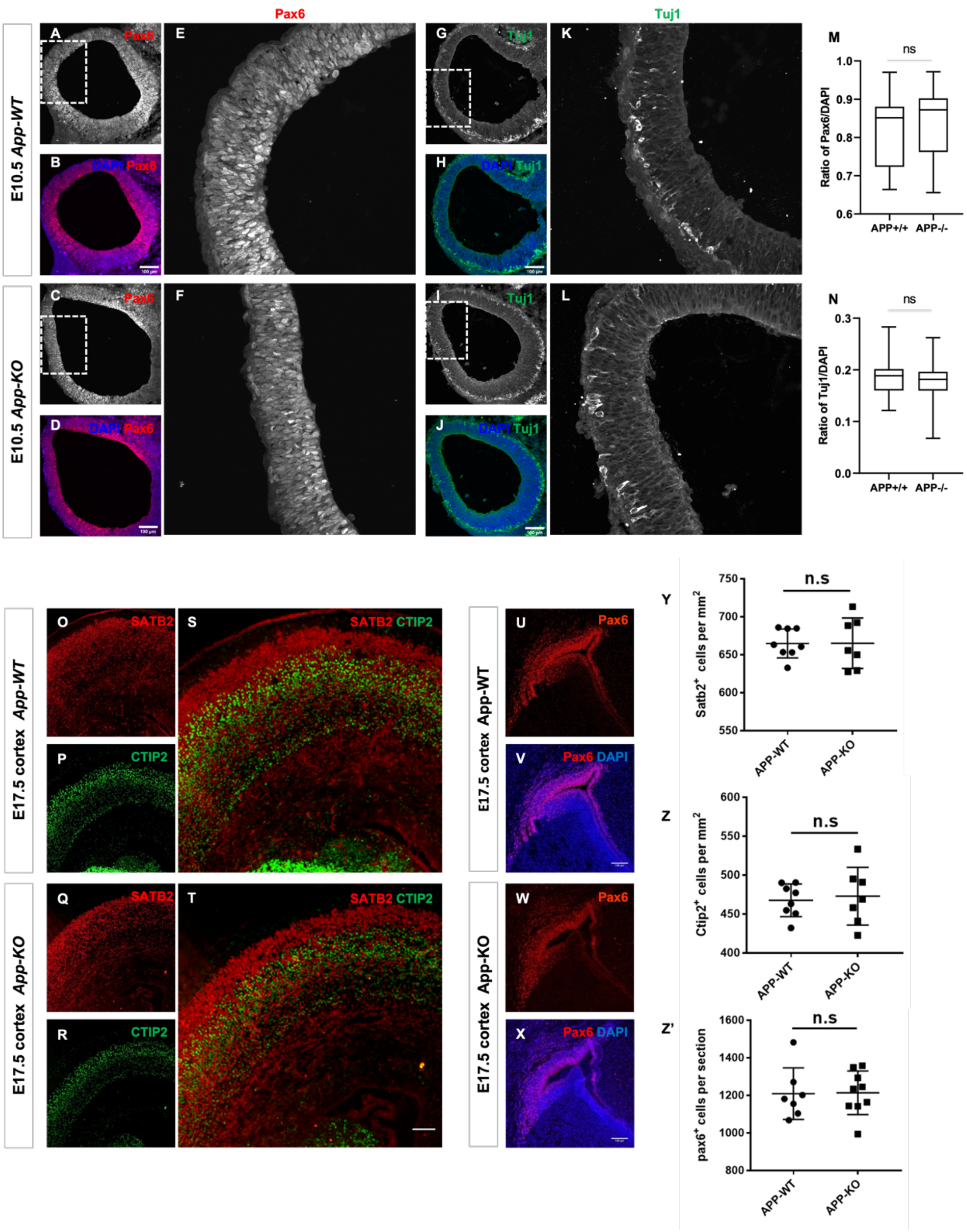

Fig. S8. Normal cell type numbers during mouse cortical neurogenesis in the absence of APP. (A-L) Brain sections of E10.5 embryo stained for Pax6 and Tuj1. (**M-N)** No difference was observed in the ratio of Pax6/DAPI and Tuj1/DAPI in *APP-WT* and *APP-KO* embryo (n=3 embryos, unpaired t-test, scale bar 100µm). **(O-X)** Brain sections of E17.5 embryo stained for Ctip2, Satb2 and Pax6. **(Y-Z')** No difference was observed in the number of Satb2+ and Ctip2+ cells (n=8 for *APP-WT* and n=7 *APP-KO,* unpaired t-test, scale bar 100µm) and number of Pax6+ (n=7 for *APP-WT* and n=9 *APP-KO*, unpaired t-test, scale bar 100µm).

Shabani-Fig. S9

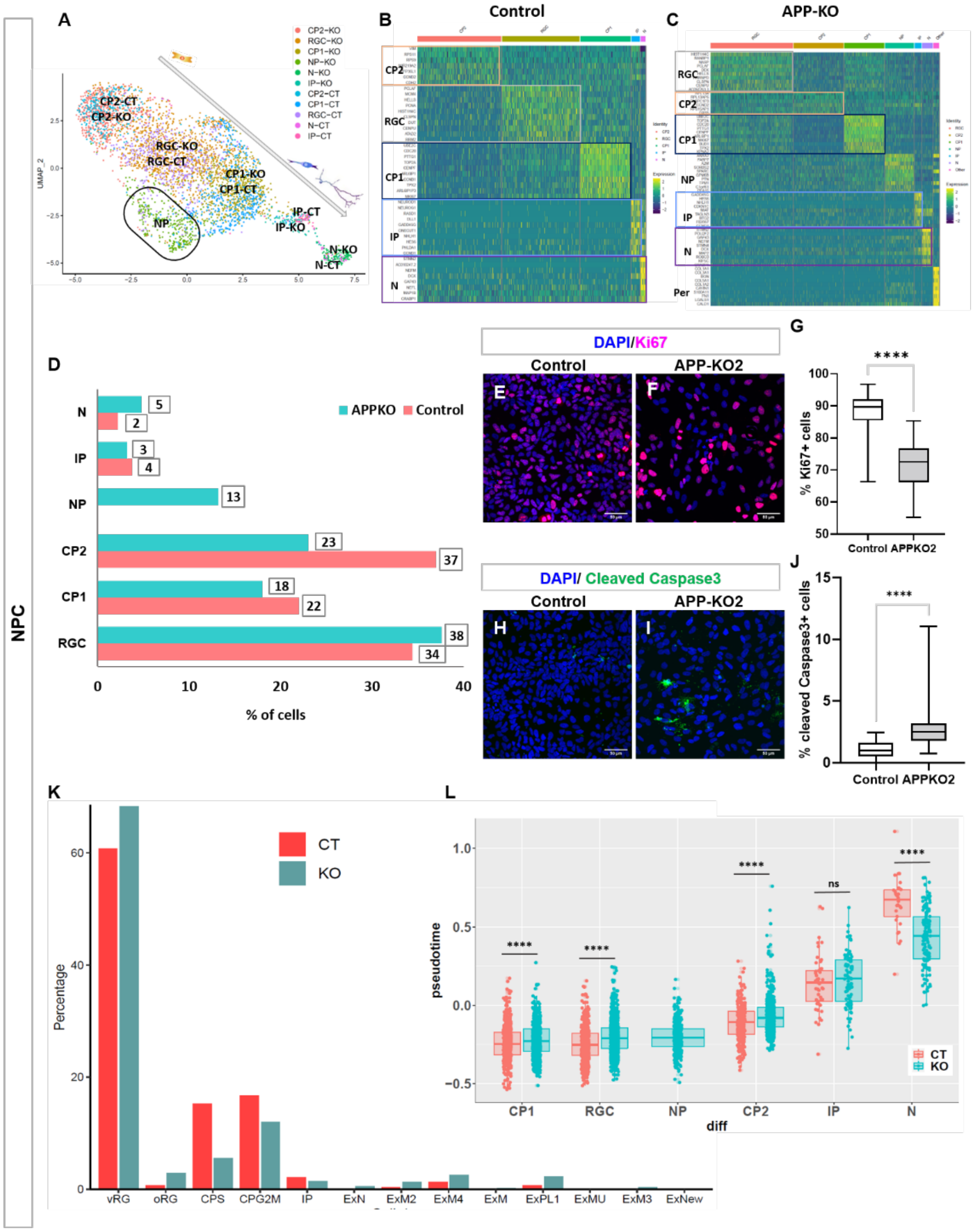

Fig. S9. ***APP-KO* NPCs are in a temporally advanced neurogenic state (A-C)** UMAP and heat maps of clustering according to the cell identity shows different clusters (RGC=radial glial cell, CP1=cycling progenitor1, CP2=cycling progenitor2, IP=intermediate progenitor, NP=neurogenic progenitor, N=neuron) in both control and *APP-KO*. **(D)** Number and percentage of cells in each cluster in isogenic control vs *APP-KO2 (***E-G)** Significant decrease in the percentage of Ki67+ cells in *APP-KO2* (n=3, unpaired t-test, p<0.0001, scale bar 50µm). **(H-J)** Significant but small increase in cell death from 1.08% in control to 2.77% in *APP-KO* shown by cleaved Caspase-3 in *APP-KO2* (n=3, unpaired t-test, p<0.0001, scale bar 50µm). **(K)** Percentage of *APP-KO* and control cells corresponding to Polioudakis et al., clustering nomenclature, upon integration with their dataset. ExNew corresponds to a cluster not identified previously. **(L)** Pseudotime analysis shows a shift toward neuronal fate in *APP-KO* clusters (p-value = 5.235e-10 for RGC, p-value = 4.554e-05 for CP2, p-value = 0.01949 for CP1, p-value = 0.1675 for IP, p-value = 1.457e-06 for neuron, Two-sample Kolmogorov-Smirnov test is used for comparison of all the clusters).

Shabani-Fig.S10

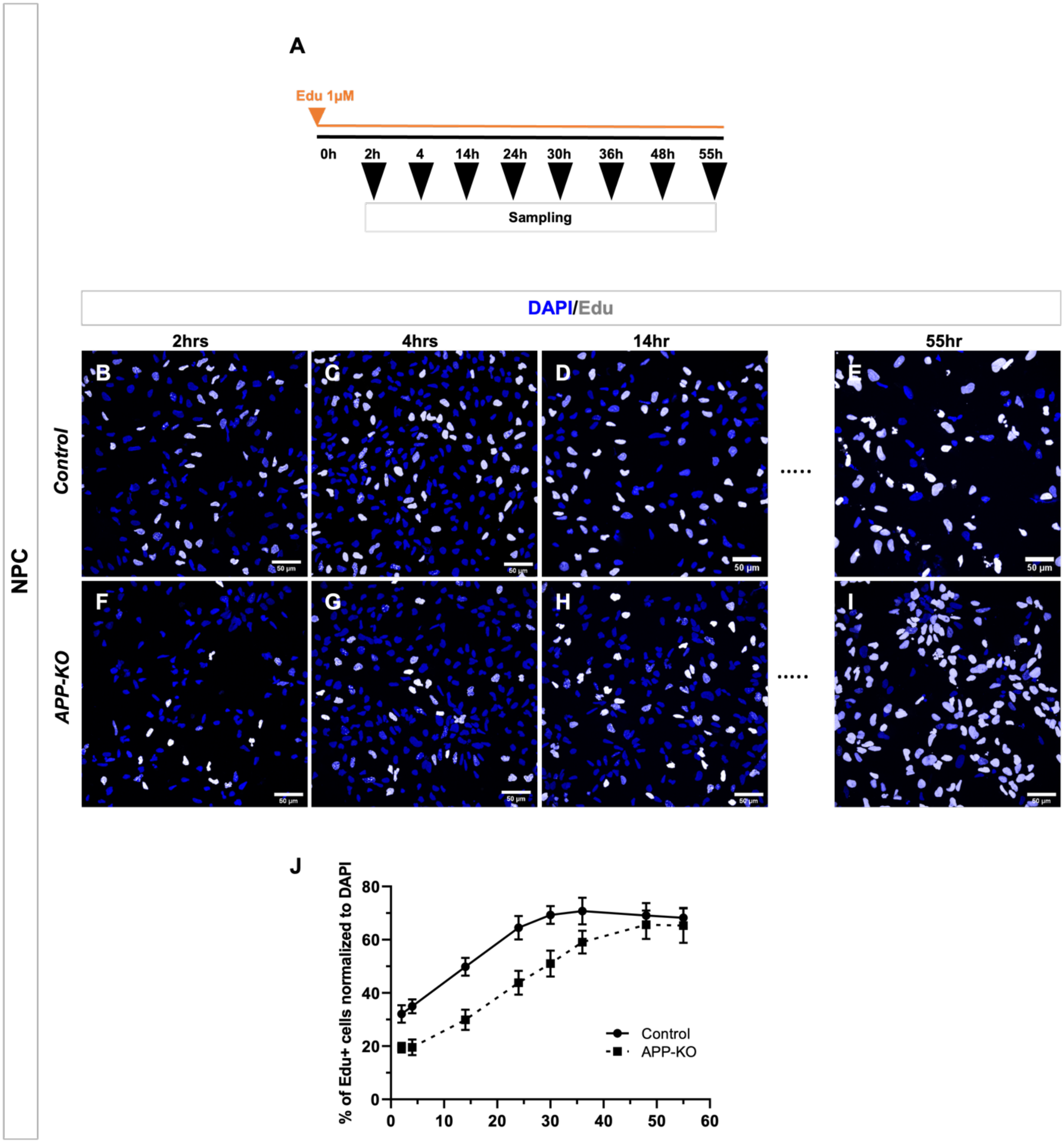

Fig. S10. APP-KO NPCs show protracted cell cycle length compare to control. (A) Experimental design of the cumulative Edu-labeling assay used to measure cell cycle length; 1µM EdU was added to the control and *APP-KO* NPC culture and kept continuously in the medium for the duration of the experiment. Cells were sampled at different time points (2, 4, 14, 24, 30, 36, 48 and 55 hours) for Edu detection. **(B-I)** Representative images of control and *APP-KO* NPC following EdU detection at 2, 4, 14 and 55 hours post sampling. **(J)** While average cell cycle length in control is 36 hours, APP-KO progenitors shows protracted cell cycle length about 48 hours. Scale bar, 50µm.

Shabani-Fig.S11

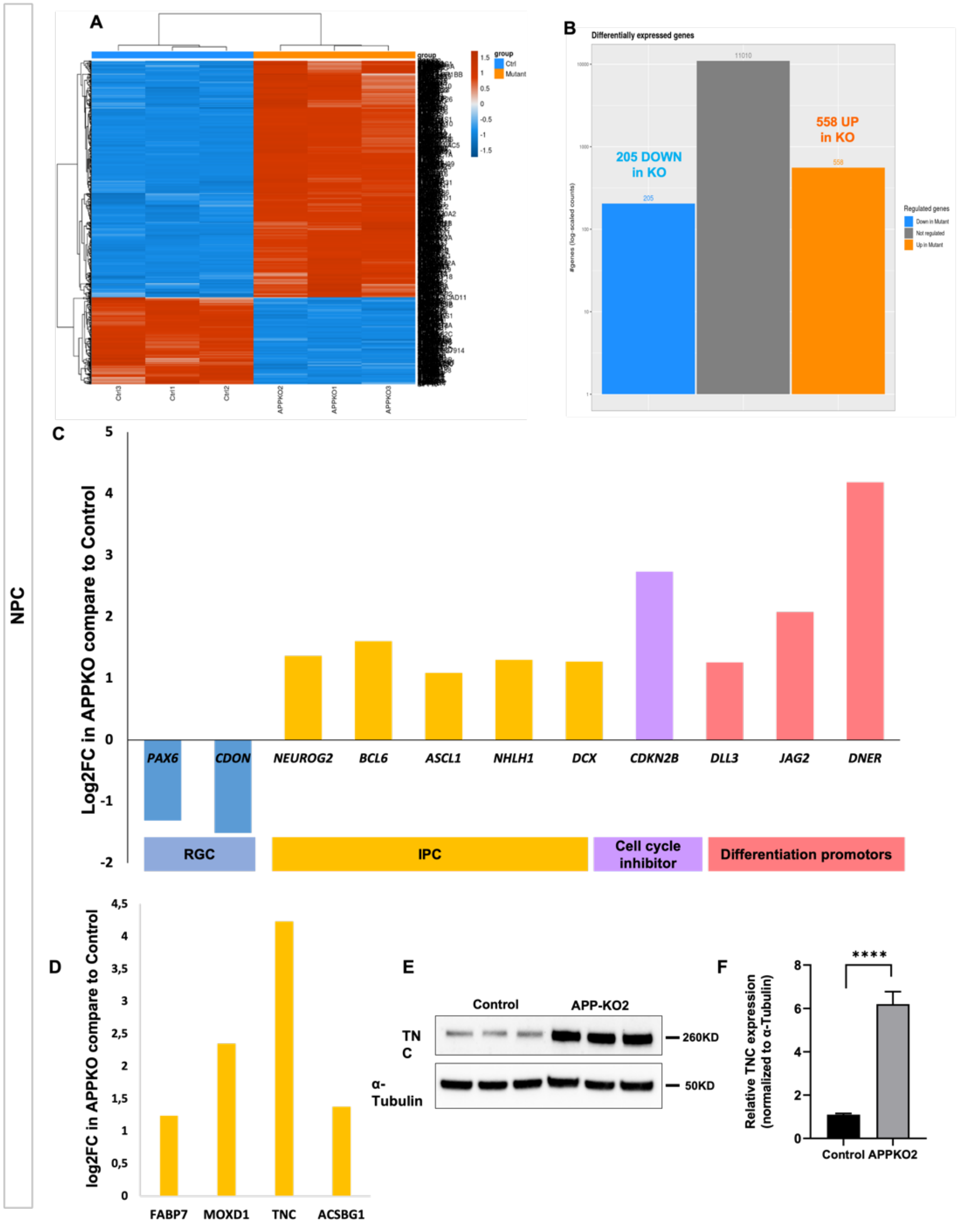

Fig. S11. Bulk RNA seq confirms the shift towards a differentiated fate in *APP-KO* NPCs. (A) Heat map of differentially expressed genes shows strikingly different patterns in isogenic control and *APP-KO2* NPCs. (*Ctrl1*,2,3 correspond to 3 technical repeats of isogenic control and *APP-KO1,2,3* correspond to 3 technical repeats of *APP-KO2*). (**B)** 763 differentially expressed genes in *APP-KO2* NPCs compared to isogenic control which 558 genes are upregulated and 205 gens are downregulated. (**C)** Bulk RNAseq shows downregulation of RGC markers (PAX6 and CDON) and upregulation of neurogenic genes (*NEUROG2*, *BCL6*, *ASCL1*, *NHLH1*, and *DCX*), cell cycle inhibitor (*CDKN2B*) and differentiation promoting Notch ligands *DLL3*, *JAG2*, *DNER*. (**D)** Upregulation of oRG enriched genes FABP7, MOXD1, TNC and ACSBG1 in *APP-KO* NPCs compare to control. **(E-F)** oRG enriched protein, TNC, shows ~6 fold increase in *APP-KO* compare to control (n=4 biologically independent repeats, unpaired t-test, P<0.0001).

Shabani-Fig.S12

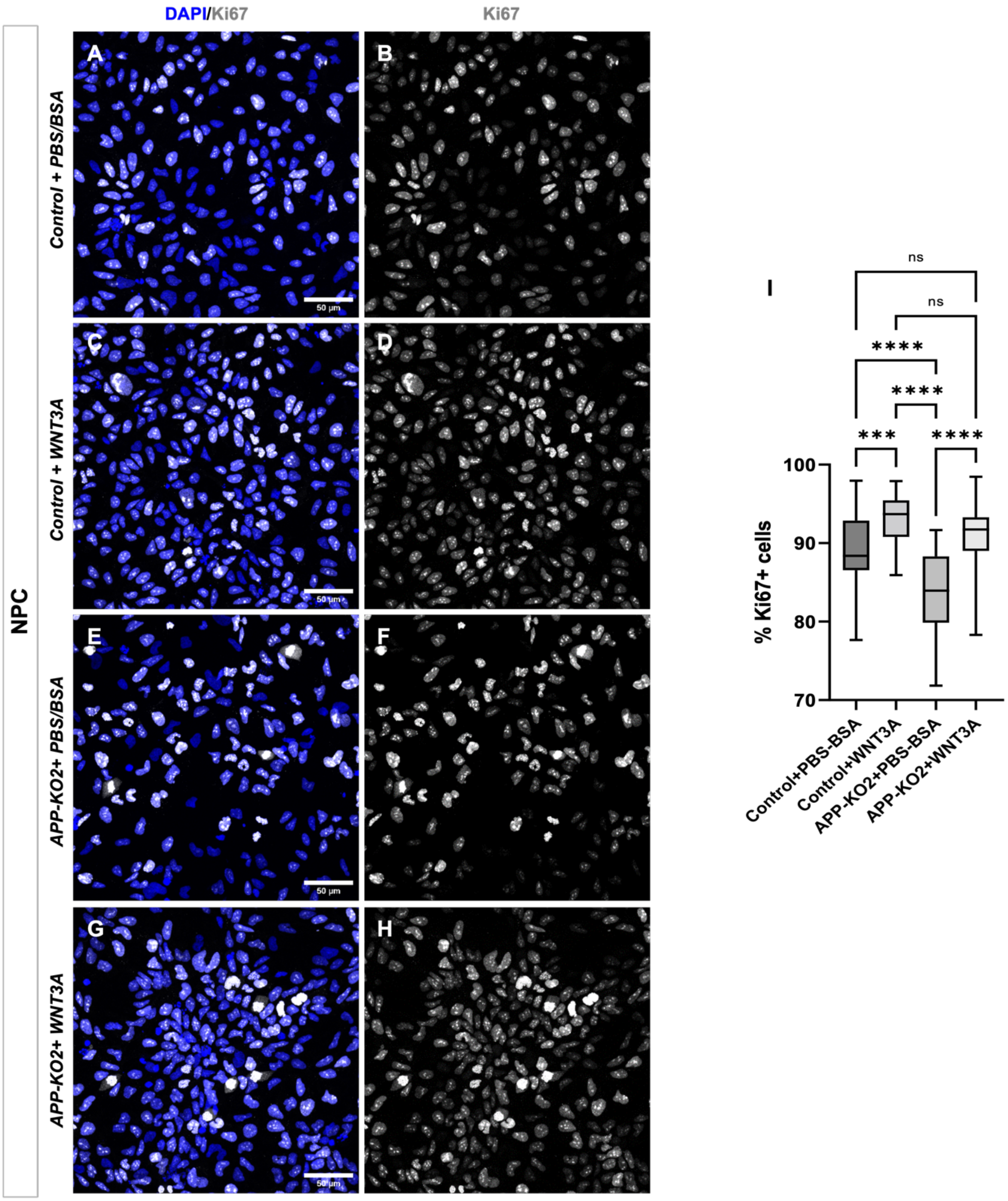

Fig. S12. Proliferation of APP KO NPCs is increased upon WNT3A treatment. **(A-H)** Isogenic control and *APP-KO2* NPCs treated with canonical WNT ligand (WNT3A) and stained for proliferation marker, Ki67. **(I)** Percentage of Ki67+ cell significantly increase in *APP-KO* background compare to control (n=3, Ordinary one-way ANOVA, p<0.0007 for isogenic control+PBS/BSA vs isogenic control+WNT3A and p<0.0001 for all the other significant conditions).

Supplementary Table 1- Number of cells per time point

	DAY0			DAY7			DAY30			DAY7/DAY0	
	**GFP**	**SOX2**	**TUJ1**	**GFP**	**SOX2**	**TUJ1**	**GFP**	**SOX2**	**TUJ1**	**No. of Progenitors generated by 1 progenitor**	**No. of Neurons generated by 1 progenitor**
**Control**	74	70	4	814	560	254	657	95	562	560/70=8	254/70=3.6
**APP-KO1**	65	52	13	860	52	808	599	15	584	52/52=1	808/52=15.5
**APP-KO2**	64	60	4	1892	106	1786	524	20	504	106/60=1.8	1786/60=29.8
**APP-KO3**	90	79	11	1868	82	1786	552	22	530	82/79=1	1786/79=22.6

Supplementary Table 2- Gene expression revealed by single cell RNA sequencing of control and APP-KO NPCs

Supplementary Table3- Gene expression revealed by bulk RNA sequencing of control and APP-KO NPCs

Supplementary FASTA file- Sequence of the Synapsin1-GFP lentiviral vector used in this study

Supplementary Table 4- Upregulated genes that have AP1 as their top transcription factor

	Cell type	Gene sign	Top 10 TF
**Progenitors**	**bRGC**	**TNC**	**AP-1** ATF-2 **c-Jun** NF-kappaB1 Sp1 STAT3
	**IPC**	**BCL6**	**AP-1** STAT5A
		**DCX**	**AP-1**
**Excitatory Neuron**	**SP**	**DKK1**	**AP-1** ATF-2 **c-Jun** GATA-6 NCX Nkx2-5
	**Deep**	**HIVEP3**	**AP-1** AREB6 ATF-2 **c-Jun** c-Myc Hlf Max Max1 Sox5 SREBP-1b
		**CAV1**	**AP-1** ATF-2 **c-Jun** IRF-1 MRF-2 Nkx2-5 Sp1 STAT3
	**UP**	**STMN2**	**AP-1** ATF-2 **c-Jun** Egr-4 NRSF form 1 NRSF form 2 Pax-5 USF-1
**Inhibitory Neuron**		**GAD1**	**AP-1** AP-2gamma ATF-2 **c-Jun** HOXA5 Pax-5
**Canonical WNT Inhibitor**		**DKK1**	**AP-1** ATF-2 **c-Jun** GATA-6 NCX Nkx2-5
		**DKK3**	**AP-1** ATF-2
		**CDKN2B**	**AP-1** p53 PPAR-gamma1 PPAR-gamma2 Sp1
**Canonical Notch**		**DNER**	**AP-1** AP-4 ATF ATF-2 **c-Jun** MyoD NRSF form 1 NRSF form 2
		**NOTCH1**	**AP-1** ATF-2 **c-Fos** **c-Jun** NF-kappaB NF-kappaB1
		**HEY1**	**AP-1** ATF-2 **c-Jun** c-Myc C/EBPalpha Evi-1 GATA-1 Max p53
**Stress response**		**NR3C1**	**AP-1**

Supplementary Table5- Guide RNAs Sequences

	Guide sequence	PAM site	Cleavage efficiency	Off target
**Guide RNA1**	GGTCGCGATGCTGCCCGGTT	TGG	8.5%	16
**Guide RNA2**	CGCAGGGTCGCGATGCTGCC	CGG	16%	74
**Guide RNA3**	TGCTGGCCGCCTGGACGGCT	CGG	3.1%	NA
**Guide RNA4**	AGCAGGAGCAGTGCCAAACC	GGG	7.4%	212

Supplementary Table 6- List of mutations in iPSC clones

16 clones homozygous mutation									
Deletion (13)							Insertion (2)		Transition (1)
6 clones	2 clones	1 clone	1 clone	1 clone	1 clone	1 clone	1 clone	1 clone	1clone
1bp	1bp	2bp	3bp	20bp	33bp	22bp	1bp	2bp	G to A
Stop codon after 40aa	Stop codon after 40 aa	Stop codon after 20aa 2 stop codon	Stop codon almost at the end of protein after 693 aa	Stop codon after 18 aa	Stop codon after 18 aa 2 stop codon	Stop codon after 18 aa	Stop codon after 22aa, 2 stop codon	Stop codon after 40 aa	Mutation in 5'-UTR, no early stop codon

Supplementary Table 7- List of Antibodies

	Antibody	Dilution	Company, Ref
Immunofluorescence	**Ki67**	1:250	Abcam ab16667
	**OCT4**	1:1000	Abcam,
	**SOX2**	1:500	Millipore, AB5603
	**SOX2**	1:20	R&D Systems, AF2018
	**PAX6**	1:200	Biolegend, PRB-278P
	**NESTIN**	1:300	Abcam, abcam 22035
	**SOX1**	1:10	R&D Systems, AF3369
	**TUJ1**	1:1000	Biolegend 802001
	**TUJ1**	1:500	Biolegend 801202
	**DCX**	1:2000	Millipore MAB2253
	**SATB2**	1:25	Abcam, ab51502
	**CTIP2**	1:1000	Abcam, 25B6
	**FOXP2**	1:500	Abcam, ab16046
	**NEUROG2**	1:200	Cell signaling Technology_ mAb 13144S
	**GFP**	1:1000	Ab13970
	**EMX2**	1:25	ab94713
	**FOXG1**	1:200	Abcam ab18259
	**ISLET1**	1:100	Abcam ab109517
	**Cleaved-Caspase3**	1:400	Cell signaling Technology 9661
Western Blot	**APP**	1:10,000	Gift from Bart de Strooper lab
	**α-TUBULIN**	1:2500	Sigma (T6199)
	**Phospho JUN**	1:1000	Cell signaling Technology 9261

Supplementary Table 8- PCR and qPCR primers

Gene	Forward Primer	Reverse primer	application
**APP**	5'-GGCTCCGTCAGTTTCCTCGG-3'	5'-CCCGGCTTCTCTGCATTAAAGA-3'	PCR
**APP**	5'-CAGAATGGGAAGTGGGATTCA-3'	5'-CAGTTCAGGGTAGACTTCTTGG-3'	qPCR
**GAPDH**	5'-TGCACCACCAACTGCTTAGC-3'	5'-GGCATGGACTGTGGTCATGAG-3'	qPCR

Supplementary movie I. 3D reconstruction of iPSC line 1 derived control cortical organoid at day 15 of culture. Control organoid stained for DAPI and Doublecortin (DCX).

Supplementary movie II. 3D reconstruction of iPSC line 1 derived *APP-KO2* cortical organoid at day 15 of culture. *APP-KO* organoid stained for DAPI and Doublecortin (DCX) showing significant increase in total DCX in *APP-KO* organoids relative to control.

Supplementary movie III. 3D reconstruction of iPSC line 2 derived control cortical organoid at day 15 of culture. Control organoid stained for DAPI, Doublecortin (DCX), and SOX2.

Supplementary movie IV. 3D reconstruction of iPSC line 2 derived *APP-KO* cortical organoid at day 15 of culture. *APP-KO* organoid stained for DAPI, Doublecortin (DCX), and SOX2 showing significant increase in total DCX in *APP-KO* organoids relative to control.
